# Single-Cell Approach to Monitor the Unfolded Protein Response During Biotechnological Processes With *Pichia pastoris*

**DOI:** 10.3389/fmicb.2019.00335

**Published:** 2019-02-27

**Authors:** Hana Raschmanová, Iwo Zamora, Martina Borčinová, Patrick Meier, Astrid Weninger, Dominik Mächler, Anton Glieder, Karel Melzoch, Zdeněk Knejzlík, Karin Kovar

**Affiliations:** ^1^Department of Biotechnology, University of Chemistry and Technology Prague, Prague, Czechia; ^2^Institute of Chemistry and Biotechnology, School of Life Sciences and Facility Management, Zurich University of Applied Sciences ZHAW, Wädenswil, Switzerland; ^3^Department of Genetics and Microbiology, Charles University, Prague, Czechia; ^4^Institute of Molecular Biotechnology, Graz University of Technology, Graz, Austria; ^5^Institute of Organic Chemistry and Biochemistry of the Czech Academy of Sciences, Prague, Czechia

**Keywords:** unfolded protein response (UPR), stress response, *Pichia pastoris*, super folder green fluorescent protein (sfGFP), flow cytometry, single-cell, fed-batch culture, heterogeneity

## Abstract

*Pichia pastoris* (*Komagataella sp*.) is broadly used for the production of secreted recombinant proteins. Due to the high rate of protein production, incorrectly folded proteins may accumulate in the endoplasmic reticulum (ER). To restore their proper folding, the cell triggers the unfolded protein response (UPR); however, if the proteins cannot be repaired, they are degraded, which impairs process productivity. Moreover, a non-producing/non-secreting subpopulation of cells might occur, which also decreases overall productivity. Therefore, an in depth understanding of intracellular protein fluxes and population heterogeneity is needed to improve productivity. Under industrially relevant cultivation conditions in bioreactors, we cultured *P. pastoris* strains producing three different recombinant proteins: penicillin G acylase from *Escherichia coli* (*Ec*PGA), lipase B from *Candida antarctica* (*Ca*LB) and xylanase A from *Thermomyces lanuginosus* (*Tl*XynA). Extracellular and intracellular product concentrations were determined, along with flow cytometry-based single-cell measurements of cell viability and the up-regulation of UPR. The cell population was distributed into four clusters, two of which were viable cells with no UPR up-regulation, differing in cell size and complexity. The other two clusters were cells with impaired viability, and cells with up-regulated UPR. Over the time course of cultivation, the distribution of the population into these four clusters changed. After 30 h of production, 60% of the cells producing *Ec*PGA, which accumulated in the cells (50–70% of the product), had up-regulated UPR, but only 13% of the cells had impaired viability. A higher proportion of cells with decreased viability was observed in strains producing *Ca*LB (20%) and *Tl*XynA (27%). The proportion of cells with up-regulated UPR in *Ca*LB-producing (35%) and *Tl*XynA-producing (30%) strains was lower in comparison to the *Ec*PGA-producing strain, and a smaller proportion of *Ca*LB and *Tl*XynA (<10%) accumulated in the cells. These data provide an insight into the development of heterogeneity in a recombinant *P. pastoris* population during a biotechnological process. A deeper understanding of the relationship between protein production/secretion and the regulation of the UPR might be utilized in bioprocess control and optimization with respect to secretion and population heterogeneity.

## Introduction

The yeast *Pichia pastoris* (*Komagataella phaffii*) is an established host for the biotechnological production of wide range of heterologous proteins, most of which are secreted (Cereghino and Cregg, 2000; Ahmad et al., 2014; Meehl and Stadheim, 2014; Juturu and Wu, 2018). The knowledge of this host and its use for the production of heterologous proteins is already advanced, but a more systematic and complex understanding of the *P. pastoris* cell factory is still needed, especially of intracellular metabolite fluxes, regulatory pathways and secretory machinery (Puxbaum et al., 2015; Zahrl et al., 2017). High-level expression of a heterologous gene may lead to an overload of the protein folding capacity of the endoplasmic reticulum (ER), and consequently to an accumulation of unfolded and/or misfolded proteins in the ER. As a response aiming to restore proper protein folding in the ER, and thus to eliminate the ER stress, the unfolded protein response (UPR) is triggered (Mattanovich et al., 2004; Guerfal et al., 2010). When the unfolded proteins cannot be repaired, they are eliminated within the ER-associated protein degradation (ERAD) pathway (Zahrl et al., 2018). The proportion of intracellularly degraded protein may be massive, up to 60% of the total (Pfeffer et al., 2011). Also, an interplay between protein synthesis and degradation to control protein homeostasis remains unclear, but was recently investigated in mammalian cells at single-cell level (Alber et al., 2018). The rate of protein degradation was shown to vary between cells (Alber et al., 2018). In a recombinant strain of *P. pastoris*, the co-existence of more sub-populations in terms of production and secretion of the recombinant protein was observed, where only part of the population actively secreted (Love et al., 2012). There was a sub-population with a stochastically changing rate of secretion (Love et al., 2010), a sub-population producing different amounts of the recombinant protein (Broger et al., 2011), and one not producing any at all (Broger et al., 2011). Intracellular protein accumulation and/or degradation, together with the existence of a non-secreting phenotype results in decreased productivity of a heterologous protein. Though an important aspect of productivity and approach to a strain's optimization, little attention is typically paid to intracellular product fluxes, stress responses (UPR, ERAD), strain heterogeneity in terms of growth and production/secretion, and cell physiology in microbial cultivation processes under industrially relevant cultivation conditions (Theron et al., 2018).

The UPR was investigated in *P. pastoris* at the point of clone selection (Aw et al., 2017), and in strains producing different recombinant proteins during fed-batch (Hohenblum et al., 2004; Resina et al., 2007; Sjöblom et al., 2012; Vogl et al., 2014; Zhong et al., 2014; Wang et al., 2017; Yu et al., 2017) or chemostat (Gasser et al., 2007; Hesketh et al., 2013; Rebnegger et al., 2014) bioreactor cultivations. Several recombinant proteins were shown to up-regulate UPR in *P. pastoris*, e.g., antibody fragments (Gasser et al., 2007; Khatri et al., 2011), human lysozyme (Hesketh et al., 2013), human trypsinogen (Hohenblum et al., 2004), lipase from *Rhizopus oryzae* (Resina et al., 2007), mucin-type protein fused with green fluorescent protein (GFP) (Sjöblom et al., 2012), membrane transporter proteins (Vogl et al., 2014), prolyl endopeptidase (Wang et al., 2017), phospholipase A_2_ from *Streptomyces violaceoruber* (Yu et al., 2017) or human interleukin (Zhong et al., 2014). In contrast, the production of human serum albumin did not lead to induction of UPR (Hohenblum et al., 2004; Aw et al., 2017).

In *P. pastoris*, the UPR has so far been studied at the level of transcript of the genes involved in the UPR, using RNAseq (Yu et al., 2017), qPCR (Guerfal et al., 2010; Khatri et al., 2011; Sjöblom et al., 2012; Zhong et al., 2014; Wang et al., 2017), TRAC (Gasser et al., 2007), microarrays (Graf et al., 2008; Dragosits et al., 2010; Rebnegger et al., 2014; Vogl et al., 2014; Edwards-Jones et al., 2015; Aw et al., 2017) or sandwich hybridization assays (Resina et al., 2007). Alternatively, the UPR was detected at the level of protein with 2D electrophoresis (Dragosits et al., 2010; Vanz et al., 2014), Western blot (Zhong et al., 2014) or flow cytometry after immunofluorescent staining (Hohenblum et al., 2004). These methods can be laborious, time-consuming and expensive. Additionally, detection of the intracellular content of mRNA or protein requires previous cell disruption, which hinders the UPR monitoring during a running bioprocess. Recently, indirect detection of UPR levels based on metabolite biomarkers was suggested. This method can be applied in a high-throughput manner, and thus is more suitable for the screening scale (Tredwell et al., 2017). In yeast cell factories other than *P. pastoris*, easily detectable reporter proteins like β-galactosidase (Cox et al., 1993; Madzak and Beckerich, 2006) or super folder green fluorescent protein (sfGFP) (Lajoie et al., 2012) were previously used to monitor the UPR.

During fed-batch bioreactor cultivations of recombinant *P. pastoris* strains producing penicillin G acylase from *Escherichia coli* (*Ec*PGA), lipase B from *Candida antarctica* (*Ca*LB) or xylanase A from *Thermomyces lanuginosus* (*Tl*XynA), we systematically analyzed the production (secretion vs. intracellular accumulation) of the recombinant protein. Moreover, with flow cytometry, i.e., at the single-cell level, at-line and in a non-invasive manner, we also analyzed the up-regulation of UPR, cell physiology and heterogeneity of the *P. pastoris* strains. To monitor the up-regulation of UPR in the *P. pastoris* strains, a plasmid bearing a gene for sfGFP under the control of the *KAR2* promoter was integrated into the *P. pastoris* genome. The sfGFP is a fast and robustly folding variant of GFP that is synthesized within a few minutes (Pédelacq et al., 2006; Khmelinskii et al., 2012), which makes it an appropriate biosensor for the immediate detection of folding events in the cell. *KAR2* is a gene involved in UPR, and its product, Kar2p protein, is an ER-resident chaperone that recognizes misfolded/unfolded proteins in the ER and assists proper protein folding (Dudek et al., 2009). Using flow cytometry for the detection of the sfGFP fluorescent signal, it was possible to monitor the activation of the *KAR2* promoter, i.e., up-regulation of the UPR at-line during the cultivation process.

## Materials and Methods

### Culture Media

YPD medium contained 20 g glucose, 20 g peptone, 10 g yeast extract and 15 g agar per liter. YPD medium with 0.1 mg mL^−1^ Zeocin™ (Invitrogen, Carlsbad, USA) was used for the selection of the transformants containing the pPICZ-α-A plasmid with different recombinant genes. YPD medium with 0.1 mg mL^−1^ Nourseothricin (Jena Bioscience, Jena, Germany) was used for the selection of the strains containing the pREP-UPS_KAR2_-sfGFP-NAT plasmid.

BMG (buffered minimal medium with glycerol) was used for screening the *P. pastoris* clones with integrated pREP-X-sfGFP-NAT or pREP-UPS_KAR2_-sfGFP-NAT plasmid and for the flask cultivation of the *P. pastoris* strain producing *Ec*PGA (section 3.3). BMG medium contained 10 g glycerol, 13.4 g yeast nitrogen base with ammonium sulfate and without amino acids (YNB), and 0.4 mg biotin per liter and 100 mM potassium phosphate buffer (pH 6.0).

BMD1% (buffered minimal medium with 1% dextrose), BMM10 and BMM2 (buffered minimal media with 5 and 1% methanol, respectively) were used for the micro-scale cultivation screening in 96-deep well plates (Weis et al., 2004). The BMD1% medium contained 10 g glucose, 13.4 g YNB and 0.4 mg biotin per liter and 200 mM potassium phosphate buffer (pH 6.0). The BMM10 medium contained 50 mL methanol, 13.4 g YNB and 0.4 mg biotin per liter and 200 mM potassium phosphate buffer (pH 6.0). The BMM2 medium contained 10 mL methanol, 13.4 g YNB and 0.4 mg biotin per liter and 200 mM potassium phosphate buffer (pH 6.0).

BMGY (buffered complex medium with glycerol) was used for growth of the inoculum for the bioreactor cultivations. BMGY medium contained 10 g glycerol, 10 g yeast extract, 20 g peptone, 13.4 g YNB and 0.4 mg biotin per liter and 100 mM potassium phosphate buffer (pH 6.0).

Defined mineral medium used for batch bioreactor cultivations contained 30 g glycerol as a sole carbon source, 2.86 g K_2_SO_4_, 0.64 g KOH, 2.32 g MgSO_4_·7H_2_O, 0.17 g CaSO_4_·2H_2_O, 0.60 g EDTA disodium dehydrate, 7.23 g 85% H_3_PO_4_, 0.22 g NaCl, 0.1 mL polypropylene glycol (PPG), 4.35 mL filter sterilized PTM1 solution and 4.35 mL filter sterilized biotin solution per liter. The PTM1 stock solution contained 5.0 mL 96% H_2_SO_4_, 3.84 g CuSO_4_, 0.08 g NaI, 3.0 g MnSO_4_·H_2_O, 0.2 g Na_2_MoO_4_·2H_2_O, 0.02 g H_3_BO_3_, 0.92 g CoCl_2_·6H_2_O, 20.0 g ZnCl_2_, and 65.0 g FeSO_4_·7H_2_O per liter. The biotin stock solution contained 0.20 g L^−1^ biotin. Before inoculation, the pH of the medium was adjusted to 5.5 using 8.5% NH_4_OH.

Concentrated medium used in the first phase of fed-batch (growth phase) contained 588 g glycerol, 12 mL of filter sterilized PTM1 solution and 12 mL of filter sterilized biotin solution per 1,000 g feed solution. Concentrated medium used in the second phase of fed-batch (production phase) contained 1,000 g methanol, 12 mL of filter sterilized PTM1 solution and 12 mL of filter sterilized biotin solution per 1,000 g feed solution.

### Plasmids

#### Construction of the UPR-Reporter Plasmid pREP-UPS_KAR2_-sfGFP-NAT

The plasmid for monitoring the UPR in recombinant *P. pastoris* strains, named pREP-UPS_KAR2_-sfGFP-NAT, carried a 324 base pair (bp) upstream region of the *KAR2* coding sequence containing one copy of the unfolded protein responsive element (UPRE) sequence, the *sfGFP* coding sequence (Khmelinskii et al., 2012), the nourseothricin acetyl transferase gene (*nat*) and the *HIS4* gene for integration of the plasmid into the *P. pastoris HIS4* locus. The construction of this plasmid is described in detail in Supplementary Figure 1. The plasmid map is provided in Supplementary Files.

#### Construction of Plasmids Bearing the Genes of the Model Recombinant Proteins

The expression cassettes for recombinant protein production contained the *AOX1* promoter, a secretion signal, the coding sequence of the heterologous gene (*Ec*PGA, *Ca*LB, *Tl*XynA), the *AOX1* terminator and the Zeocin resistance cassette. In the case of *Ec*PGA and *Ca*LB, the α-factor secretion signal from *S. cerevisiae* was used as the secretion signal, and in the case of *Tl*XynA, the natural secretion signal from *Thermomyces lanuginosus* was kept (Mellitzer et al., 2012b).

Construction of the expression plasmid carrying the *Ec*PGA gene (Sobotková et al., 1996) (pPICZαA_EcPGA) is described elsewhere (Borcinova et al., in preparation). Briefly, the coding sequence of the *Ec*PGA gene was codon optimized for *P. pastoris* and cloned into the pPICZα A plasmid (Invitrogen, Carlsbad, USA) via *XhoI* and *XbaI* restriction sites. The coding sequence of the *Ca*LB gene, codon optimized for *P. pastoris*, was PCR-amplified from the plasmid pBSY3S1Z_CaLB-WT (bisy e.U., Hofstätten an der Raab, Austria) using primers alphaMF-CALB_fw and CALB-AOX1TT_rev, and cloned into pBSYAOXsec_blunt containing the α-factor secretion signal from *S. cerevisiae* (bisy e.U., Hofstätten an der Raab, Austria) via *MlyI* restriction sites. The constructed plasmid was named pBSYAOXsec_CaLB. The natural secretion signal and the coding sequence of *Tl*XynA, codon optimized for *P. pastoris*, were PCR-amplified from the gBlock XylA_opt using primers PAOX1-XYL_fw and XYL-AOX1TT_rev, and cloned into pBSYAOX_blunt (bisy e.U., Hofstätten an der Raab, Austria) via *MlyI* restriction sites. The constructed plasmid was named pBSYAOX_TlXynA.

The nucleotide sequences of all the above-mentioned primers are provided in Supplementary Table 1. The plasmid maps are provided in Supplementary Files.

### Strains

Electro-competent *P. pastoris* X33 (Invitrogen) cells were prepared and transformed [according to the protocol described by Lin-Cereghino et al. (2005)] with the plasmid DNA and the cells were regenerated in 1 mL of a 1:2 mixture of 1 M sorbitol and YPD medium for 2 h. For the transformation of *P. pastoris* cells with the UPR-reporter or -control plasmid, ~500 ng of *SalI*-linearized plasmid pREP-UPS_KAR2_-sfGFP-NAT or pREP-X-sfGFP-NAT was used. The transformants were plated out on selective YPD-agar plates with 0.1 mg mL^−1^ nourseothricin (Jena Bioscience, Jena, Germany) and incubated for 2–3 days at 28°C. Selected transformants were cultured overnight in 2 mL BMG at 250 rpm and 28°C, and the production of sfGFP was analyzed by flow cytometry (see chapter Flow Cytometry). An average-expressing clone from each construct was used for further studies. For the transformation of *P. pastoris* cells with the plasmid carrying the *Ec*PGA gene, pPICZαA_EcPGA, ~5 μg of the linearized plasmid DNA were used and the transformants were selected on YPD-agar plates with Zeocin (Invitrogen, Carlsbad, USA) (Borcinova et al., in preparation). For the transformation of *P. pastoris* cells with the plasmids carrying the *Ca*LB or *Tl*XynA genes, ~2 μg of *PmeI*-linearized plasmid pBSYAOXsec_CaLB or pBSYAOX_TlXynA, respectively were used for the transformation. The transformants were plated out on selective YPD-agar plates with 0.1 mg mL^−1^ Zeocin (Invitrogen, Carlsbad, USA) and incubated for 2–3 days at 28°C. Random transformants were screened in 96-deep well plates (see chapter Micro-Scale Cultivation Screening). All strains used in this study were stored in 24% glycerol at −80°C.

### Micro-Scale Cultivation Screening

*P. pastoris* clones producing *Ca*LB or *Tl*XynA were cultured in 96-deep well plates in shakers (INFORS Multitron, Bottmingen, Switzerland) at 28°C, 320 rpm, and 80% relative humidity (Weis et al., 2004). After an initial batch phase for 60 h in 250 μL BMD1%, the cultures were induced with addition of 250 μL BMM2, i.e., 0.5% methanol. After 12, 24, and 48 h from the first induction, 50 μL of BMM10 was added to keep the methanol concentration at app. 0.5%. After 60 h from the first induction with methanol, the culture was centrifuged (4,000 rpm, 15 min, 4°C) and the enzymatic activity of *Ca*LB or *Tl*XynA was determined in the supernatant according to the protocols described in chapter Protein Analysis. From each construct, the clone with the highest enzymatic activity, irrespective of the number of integrated gene copies (not determined), was used for further studies.

### Bioreactor Cultivations

#### Batch Cultivations for Determination of Growth Characteristics

A glycerol stock (1 mL) of the strain (all listed in Table 1) was thawed and used to inoculate 100 mL of BMGY medium. This first seed culture was grown for 48 h at 30°C and 150 rpm, and then 20 mL of the culture were used to inoculate 180 mL of sterile BMGY (i.e., 10% inoculation ratio). This second seed culture was grown for 12 h at 30°C and 150 rpm, and then aseptically transferred to a 3.6-liter bioreactor (Infors AG, Bottmingen, Switzerland) to achieve a 10% inoculation ratio. The batch volume was 2 L. After glycerol (30 g per liter) was consumed, i.e., after the end of glycerol batch, a batch phase with methanol was performed, which was initiated by pulsing 15 g of methanol per liter to the culture. The maximum specific growth rate of biomass and biomass/substrate yield with glycerol and methanol were calculated from the data obtained from the batch cultivation with glycerol and methanol, respectively (see chapter Data Analysis) for each strain, and were comparable for all strains (Supplementary Table 2).

**Table 1:** Overview of the fed-batch cultivations.

**Recombinant protein**	**None**		***Ec*****PGA**		***Ca*****LB**			***Tl*XynA**
Strain	Pp1 (control)	Pp1 (control)	Pp4	Pp5 (control)	Pp10	Pp10	Pp10	Pp14
*sfGFP* expression	*P_*KAR*2_*	*P_*KAR*2_*	*P_*KAR*2_*	No promoter (x)	*P_*KAR*2_*	*P_*KAR*2_*	*P_*KAR*2_*	*P_*KAR*2_*
μ_methanol_ (h^−1^) set	0.016	0.032	0.016	0.016	0.016	0.032	0.008	0.016
μ_methanol_ (h^−1^) reached	0.021 ± 0.001	0.039 ± 0.001	0.019 ± 0.002	0.021 ± 0.002	0.018 ± 0.001	0.036 ± 0.001	0.007 ± 0.001	0.019 ± 0.001

*P. pastoris strains producing three different recombinant proteins and/or containing a reporter of UPR up-regulation were constructed. The recombinant proteins were EcPGA, CaLB, and TlXynA. The reporter of the up-regulation of UPR was a genome-integrated cassette consisting of the KAR2 promoter and sfGFP gene. Three strains contained the UPR reporter and produced a recombinant protein—EcPGA (Pp4), CaLB (Pp10), or TlXynA (Pp14). A control strain to verify the suitability of our UPR reporter produced EcPGA and contained a genome-integrated cassette carrying the sfGFP gene, but no promoter in front of this gene (Pp5). A control strain to examine the UPR up-regulation by cultivation conditions contained the UPR reporter, but produced none of the three recombinant proteins of interest (Pp1). In all processes, temperature was maintained at 30°C and pH at 5.5. All processes consisted of a batch phase with glycerol (30 g l^−1^), a short (2.5 h) growth fed-batch with an exponential feeding of glycerol to maintain the specific growth rate at 0.2 h^−1^, and a production fed-batch with an exponential feeding of methanol to maintain the specific growth rate at 0.008, 0.016, or 0.032 h^−1^*.

#### Fed-Batch Cultivations

A glycerol stock (1 mL) of the strain (all listed in Table 1) was thawed and used to inoculate 100 mL of BMGY medium. This first seed culture was grown for 48 h at 30°C and 150 rpm, and then 60 mL of the culture were used to inoculate 540 mL of sterile BMGY (i.e., 10% inoculation ratio). This second seed culture was grown for 12 h at 30°C and 150 rpm, and then aseptically transferred to an 18-liter bioreactor (Bilfinger Industrietechnik, Salzburg, Austria) to achieve a 10% inoculation ratio. All cultivations were carried out as single experiments (i.e., without replication) at 30°C, pH 5.5, 0.5 bar overpressure, 3 VVM airflow (without oxygen enrichment) and agitation speed 1,100 rpm. Ammonium hydroxide (8.5%) and phosphoric acid (8.5%) were used for pH adjustment. The batch volume was 6 L and the end of batch was indicated by a rapid increase in dissolved oxygen concentration in the medium (after ~12 h). To increase the biomass concentration, exponential feeding of a concentrated glycerol solution (58.8% w/w glycerol) was immediately initiated in order to keep specific growth rate μ at a constant value of 0.180 h^−1^ (app. 80% μ_max_ with glycerol) according to the feeding equation *F* = 61.88·*e*^0.18·*t*^. After 2.5 h of glycerol feeding, exponential feeding of a concentrated methanol solution (100% w/w methanol) was initiated to keep the specific growth rate μ at a constant value of 0.008 h^−1^ (app. 15% μ_max_ with methanol), 0.016 h^−1^ (app. 30% μ_max_ with methanol) or 0.032 h^−1^ (app. 60% μ_max_ with methanol). The respective feeding equations were *F* = 7.56·*e*^0.008·*t*^, *F* = 12.52·*e*^0.016·*t*^ and *F* = 22.44·*e*^0.032·*t*^. The feeding rates were calculated according to equation (3) in chapter Data Analysis. As parameters μ_max_ and *Y*_*x*/*s*_,_max_ in equation (3), the average value of the maximum specific growth rates and maximum biomass/substrate yields of the strains Pp4 and Pp5 was used (since these strains were cultured as the first ones in the fed-batch cultivations). Samples were withdrawn regularly during fed-batch culture.

### Substrate Analyses

The concentrations of glycerol and methanol in the centrifuged cultivation medium (14,000 rpm, 5 min) were determined by high-pressure liquid chromatography (HPLC) using an LC-20AB device equipped with autosampler SIL-20A, thermostated column oven CTO-20A and refractometer detector RID-10A (all produced by Shimadzu). The Aminex HPX-87H column, with an internal diameter (i.d.) of 7.8 mm (Bio-Rad, Munich, Germany), was run at 40°C at a flow rate of 0.6 mL min^−1^ under isocratic conditions, with 2.5 mM H_2_SO_4_ and an injection volume of 25 μL.

### Biomass Concentration

The cells were harvested by centrifugation (14,000 rpm, 5 min), washed with distilled water and dried at 105°C until constant weight. The biomass concentration was then determined as cell dry weight (CDW).

### Flow Cytometry

A BD Accuri™ C6 flow cytometer (BD Biosciences, Franklin Lakes, USA) equipped with a 20 mW 488 nm solid state blue laser was employed for measurements of the green fluorescence of sfGFP (FL1 530 ± 15 nm) during the flask experiments for the characterization of the *KAR2* upstream region (chapter Development of the UPR-Reporter Based on Characterization of the *KAR2* Upstream Region) and the for the characterization of the population producing recombinant *Ec*PGA (chapter Evaluation of the Flow Cytometry Data From Bioreactor Cultivations). Prior to analysis, cells were centrifuged (2,000 g, 5 min) and resuspended in phosphate-buffered saline (PBS) to an OD_600_ of 0.5. The data were analyzed using BD Accuri CFlow® Plus software (BD Biosciences, Franklin Lakes, USA).

Cells from the bioreactor experiments (chapters Evaluation of the Flow Cytometry Data From Bioreactor Cultivations and Physiology- and UPR-Related Effects of Production of Different Heterologous Proteins) were stained with propidium iodide (PI, Invitrogen, 1.0 mg/mL solution in water, P3566) and analyzed using a BD FACSCalibur 4CA flow cytometer (Becton Dickinson GmbH, Heidelberg, Germany) equipped with a 488-nm argon-ion laser, a 635-nm red diode laser, and the appropriate FACSFlow sheath fluid. Propidium iodide was used to assess cell viability as integrity of the cytoplasmic membrane, since PI effectively only enters cells with non-intact or damaged membranes and intercalates into double-stranded nucleic acids, resulting in a red fluorescence; such cells were considered as non-viable. The fluorescence emitted was collected in two optical channels, FL1 (515–545 nm) for the green fluorescence of sfGFP and FL3 (>650 nm) for the red fluorescence of propidium iodide. Samples were taken directly from the culture and were diluted with phosphate-buffered saline (PBS) to reach < 100,000 events per 30 s analysis at a low flow speed (12 μL min^−1^). The diluted *P. pastoris* cell suspension was incubated in the BD measuring tube with PI protected from light, and was thus measured directly. BD CellQuest Pro software was used in the cytometer, while the data were analyzed using FlowJo software (Tree Star Inc.).

The flow cytometry data from the bioreactor cultivations (chapters Evaluation of the Flow Cytometry Data From Bioreactor Cultivations, Physiology- and UPR-Related Effects of Production of Different Heterologous Proteins, and The Influence of Specific Growth Rate on *Ca*LB Production, Physiology and ER-Stress) were evaluated in RStudio, using principle component analysis (PCA) in order to reduce redundancy (e.g., cell size-based effects on sfGFP content). With PCA, the natural logarithm (ln) values of the measured flow cytometric data (FSC, SSC, FL1, FL2, FL3) were de-correlated and displayed in a two dimensional system of two principle components (PCs). The combination PC3-PC2, which described about 39.8% of the population variability, showed the lowest level of redundancy and was used for a clustering analysis. The clustering algorithm was developed for a sample of 10,000 measured cells from three different processes (cultivations of the strains producing *Ec*PGA and *Ca*LB, and the non-producing strain with μ_methanol_ 0.016 h^−1^), using k-means. The number of clusters was allowed to vary from one to six in order to maximize the resulting Dunn index. The remaining measurements were then clustered according to the determined population centers, using a nearest neighbors approach.

### Fluorescent Microscopy

For microscopic examination of cell size, morphology and fluorescence, phase contrast and epi-fluorescence microscopy were employed using an Olympus BX51 microscope (Olympus, Tokyo, Japan) equipped with a 120 W mercury vapor arc lamp and a U-MWB2 filter cube (excitation 450–480 nm, emission >515 nm). The microphotographs in Supplementary Figure 6 were shot directly by camera (EOS 600D, Cannon, Tokyo, Japan).

### Protein Analysis

#### Determination of Total Protein Concentration

Quick Start™ Bradford 1x Dye Reagent (Bio-Rad, Hercules, USA) was used for the determination of the total protein concentration (mg L^−1^). The assay was performed according to the producer's instructions. Briefly, 250 μL of the reagent was mixed with 5 μL of the centrifuged supernatant (14,000 rpm, 5 min) in a 96-well plate, the plate was incubated for 5 min at room temperature and then the absorbance at 595 nm was measured. Bovine gamma-globulin (part of the kit) was used as a standard.

#### Penicillin G Acylase Activity Assay

The enzymatic activity of PGA was determined by measuring the amount of 6-APA generated by hydrolysis of penicillin G (Gao et al., 2006). 50–300 μL of the centrifuged supernatant (14,000 rpm, 5 min) were mixed with 100 mM sodium phosphate buffer (pH 8.0) to reach a total volume of 2 mL. Then, 1 mL of substrate solution (2% (w/v) penicillin G K^+^ salt in 100 mM sodium phosphate buffer, pH 8.0) was added and the reaction mixture was incubated at 37°C for 8 min. In 2, 4 and 8 min after the addition of the substrate, 0.5 mL of the reaction mixture was withdrawn and the reaction was immediately stopped by the addition of 3 mL stop solution [mixture of 20% (v/v) acetic acid and 50 mM NaOH in ratio 2:1 (v/v)]. Then, 0.5 mL of p-dimethylamidobenzaldehyde solution in methanol (5 g L^−1^) was added to the stopped reaction. After 15 min incubation of the mixture at room temperature, its absorbance at 415 nm was measured. One activity unit of PGA (U) is defined as the amount of PGA that is needed to produce 1 μmol 6-APA mL^−1^ min^−1^.

For measurement of the intracellular *Ec*PGA, centrifuged cells from a 4 mL sample were washed with 0.1 M sodium phosphate buffer (pH 8.0) and stored at −80°C for a minimum of 1 h. The cell pellet was then resuspended to the original volume (4 mL) and diluted with 0.1 M sodium phosphate buffer (pH 8.0), to reach a maximum absorbance OD_600_ of 50–100. 1 mL of the diluted cell suspension was mixed with 1 mL of glass beads (0.25–0.50 mm) and vortexed for 20 min. The vortexed sample was centrifuged (8,000 rpm, 5 min, 4°C) and the supernatant was used for the PGA activity assay.

#### Lipase B Activity Assay

The enzymatic activity of *Candida antarctica* lipase B (*Ca*LB) was determined according to the colorimetric assay using p-nitrophenyl butyrate (pNPB) as a substrate (Krainer et al., 2012). 20 μL of the enzyme sample (culture supernatant) was transferred into a 96-well microtiter plate, and 180 μL of a freshly prepared pNPB working solution (100 μL pNPB stock solution in 10 mL of 300 mM Tris/HCl buffer, pH 8.0; pNPB stock solution was prepared by mixing 42 μL of pNPB (Sigma Aldrich) with 458 μL of DMSO and stored at −20°C) was added. The kinetics of the reaction were measured immediately at 405 nm for 6 min at 25°C. One activity unit (U) of *Ca*LB was defined as the amount of *Ca*LB that was needed to produce 1 μmol p-nitrophenolate mL^−1^ min^−1^.

For the measurement of the intracellular *Ca*LB, centrifuged cells from a 0.5 mL sample were washed with PBS and stored at −20°C. The cell pellet was gently resuspended in Y-PER™ Yeast Protein Extraction Reagent (Thermo Scientific, Rockford, USA) in ratio 2.5 μL Y-PER per 1 mg WCW. The suspension was then incubated in the Eppendorf agitator at room temperature for 30 min and 600 rpm. Then, the sample was centrifuged (14,000 rpm, 10 min) and the supernatant was used for the *Ca*LB activity assay.

#### Xylanase a Activity Assay

The enzymatic activity of *Thermomyces lanuginosus* (*Tl*XynA) was determined according to the colorimetric *para*-hydroxybenzoic acid hydrazide (pHBAH) assay, which is used to detect reducing sugars released from polymers (Mellitzer et al., 2012a). For lignocellulosic substrate conversion, 20 μL of the enzyme sample (culture supernatant) or blank (50 mM sodium citrate buffer, pH 4.8) was mixed with 150 μL of the substrate solution (0.5% xylan in 50 mM sodium citrate buffer, pH 4.8). The mixture was incubated for 30 min at 50°C and 300 rpm. For the subsequent reducing-sugar assay, 50 μL of the substrate conversion reaction was mixed with 150 μL of pHBAH working solution (pHBAH stock solution was 5% w/v pHBAH in 0.5% v/v HCl; pHBAH working solution was prepared by mixing the stock solution with 0.5 M NaOH in 1:4 v/v ratio). The mixture was incubated at 99°C for 2 min, and then cooled to 4°C. Then, the absorption at 410 nm was measured. The activity (U L^−1^) of *Tl*XynA was calculated from a standard curve, which was obtained after performing the assay with a series of standard solutions (0–200 U L^−1^) of commercial xylanase from *Thermomyces lanuginosus* (X2753, Sigma Aldrich, St. Louis, USA).

For the measurement of intracellular *Tl*XynA, centrifuged cells from a 0.5 mL sample were washed with PBS and stored at −20°C. The cell pellet was gently resuspended in Y-PER™ Yeast Protein Extraction Reagent (Thermo Scientific, Rockford, USA) in ratio 2.5 μL Y-PER per 1 mg WCW. The suspension was then incubated in the Eppendorf agitator at room temperature for 30 min and 600 rpm. Then, the sample was centrifuged (14,000 rpm, 10 min) and the supernatant was used for the *Tl*XynA activity assay.

### Fluorimetric Measurements

Spark 20M multimode microplate reader (Tecan, Männedorf, Switzerland) was used for measuring fluorescence of sfGFP. The mode Fluorescence Top Reading was used, with the excitation wavelength 485 ± 10 nm and emission wavelength 535 ± 10 nm.

### qPCR

The sample taken during the bioreactor cultivation was centrifuged (14,000 rpm, 5 min) and the cell pellet was stored at −80°C. Total RNA was isolated from the cell pellets according to the protocol provided by Invitrogen (http://tools.thermofisher.com/content/sfs/manuals/easyselect_man.pdf). The cDNA was prepared using the iScript cDNA Synthesis Kit (Bio-Rad, Hercules, USA). qPCR was performed in QuantStudio™ 5 Real-Time PCR System (ThermoFisher Scientific, Waltham, USA), using the 5 × HOT FIREPol® EvaGreen® qPCR Mix Plus (ROX) (Solis BioDyne, Tartu, Estonia). Relative expression of *KAR2* was monitored (primers q34 and q35), using *ACT1* as a reference (primers q24 and q25) and no-template as well as no-RT controls. The nucleotide sequences of the primers are provided in Supplementary Table 1. The fold-change of *KAR2* expression was evaluated using the ΔΔc_t_ method, relating all expression data to the expression of *KAR2* in the non-producing control strain in the sample taken 3 h after induction.

### Statistical Analysis and Controls

Measurements of biomass concentration were carried out in duplicate. Flow cytometric measurements, measurements of enzyme activity and total protein concentration, and qPCR measurements were carried out in triplicate. The mean values was calculated and the errors were expressed as standard deviations.

### Data Analysis

Maximum specific growth rate was calculated according to equation (1):

(1) $$\begin{array}{cc} \mu_{\max}=\frac{\ln(x(t))-\ln(x_{0})}{t-t_{0}} & \begin{array}{c} \left({\text{h}}^{-1}\right) \end{array} \end{array}$$

where *x* is the biomass concentration (g L^−1^) and *t* is the cultivation time (h).

Maximum biomass/substrate yield was calculated according to equation (2):

(2) $$\begin{array}{cc} Y_{x/s,\max}=\frac{x_{2}-x_{1}}{s_{2}-s_{1}} & \begin{array}{c} \left(\text{g}\;{\text{g}}^{-1}\right) \end{array} \end{array}$$

where *s* is the substrate concentration (g L^−1^).

Exponential feeding rate was calculated according to equation (3):

(3) $$\begin{array}{cc} F=F_{0}\cdot e^{\mu \cdot t}=\frac{\mu \;\cdot \;V_{0}\;\cdot \;x_{0}}{Y_{x/s,\max}\;\cdot \;s_{0}}\cdot e^{\mu \cdot t} & \begin{array}{c} \left(\text{L}\;{\text{h}}^{-1}\right) \end{array} \end{array}$$

where *F*_0_ is the initial feed rate (L h^−1^), μ is the required specific growth rate (h^−1^), *V*_0_ is the initial working volume (L), *x*_0_ is the initial biomass concentration (g L^−1^) and *s*_0_ is the concentration of carbon source in the feed solution (g L^−1^).

Specific growth rate during fed-batch was calculated according to equation (4):

(4) $$\begin{array}{cc} \mu (t)=\frac{\ln\left(x\;\cdot \;V\right)-\ln\left(x_{0}\;\cdot \;V_{0}\right)}{t-t_{0}} & \begin{array}{c} \left({\text{h}}^{-1}\right) \end{array} \end{array}$$

where *V* is the working volume (L).

Approximate volume of supernatant was calculated according to equation (5):

(5) $$\begin{array}{cc} V_{s}(t)=\frac{\left(V\left(t\right)\;\cdot \;\rho_{\text{broth}}\right)-\frac{M_{x.\;\;WCW}}{1000}}{\rho_{\text{supernatant}}} & \begin{array}{c} \left(\text{L}\right) \end{array} \end{array}$$

where ρ_broth_ is the density of culture broth (kg L^−1^), *M*_*x*__, WCW_ is the mass of wet cells (g L^−1^) and ρ_supernatant_ is the density of supernatant that is ~1.03 kg L^−1^ (Potgieter et al., 2010)

The density of culture broth was calculated according to equation (6):

(6) $$\begin{array}{cc} \rho_{\text{broth}}\approx 0.000132\;\cdot \;x_{\text{WCW}}+1.03 & \begin{array}{c} \left({\text{kg L}}^{-1}\right) \end{array} \end{array}$$

where *x*_WCW_ is the concentration of biomass wet cell weight (g L^−1^).

The concentration of wet biomass (g L^−1^) was calculated according to equation (7). This equation was determined and verified experimentally for the given strain and culture conditions.

(7) $$\begin{array}{cc} \text{WCW}\approx 4.2\cdot \text{CDW} & \left({\text{g L}}^{-1}\right). \end{array}$$

Product/biomass yield during fed-batch was calculated according to equation (8):

(8) $$\begin{array}{cc} Y_{p/x}=\frac{\Delta \left(c_{p}\cdot V_{S}\right)}{\Delta \left(x\cdot V\right)} & \begin{array}{c} \left({\text{U g}}^{-1}\right) \end{array} \end{array}$$

where *c*_p_ is the product concentration (U L^−1^).

## Results

We constructed a reporter for monitoring UPR up-regulation in living *P. pastoris* cells, which was based on the production of sfGFP upon the activation of the *P. pastoris KAR2* promoter. Using this reporter, UPR up-regulation was assessed in *P. pastoris* strains producing different recombinant proteins (*Ec*PGA, *Ca*LB, *Tl*XynA) during bioreactor fed-batch cultivations with methanol. Besides assessing the concentration of the secreted product in the cultivation medium, we also measured the residual/non-secreted product inside the cells. Flow cytometry was used as an at-line, single-cell and non-invasive method for the assessment of the UPR up-regulation (of the fluorescence of sfGFP), cell viability and population heterogeneity.

### Development of the UPR-Reporter Based on Characterization of the *KAR2* Upstream Region

**Figure 1:**
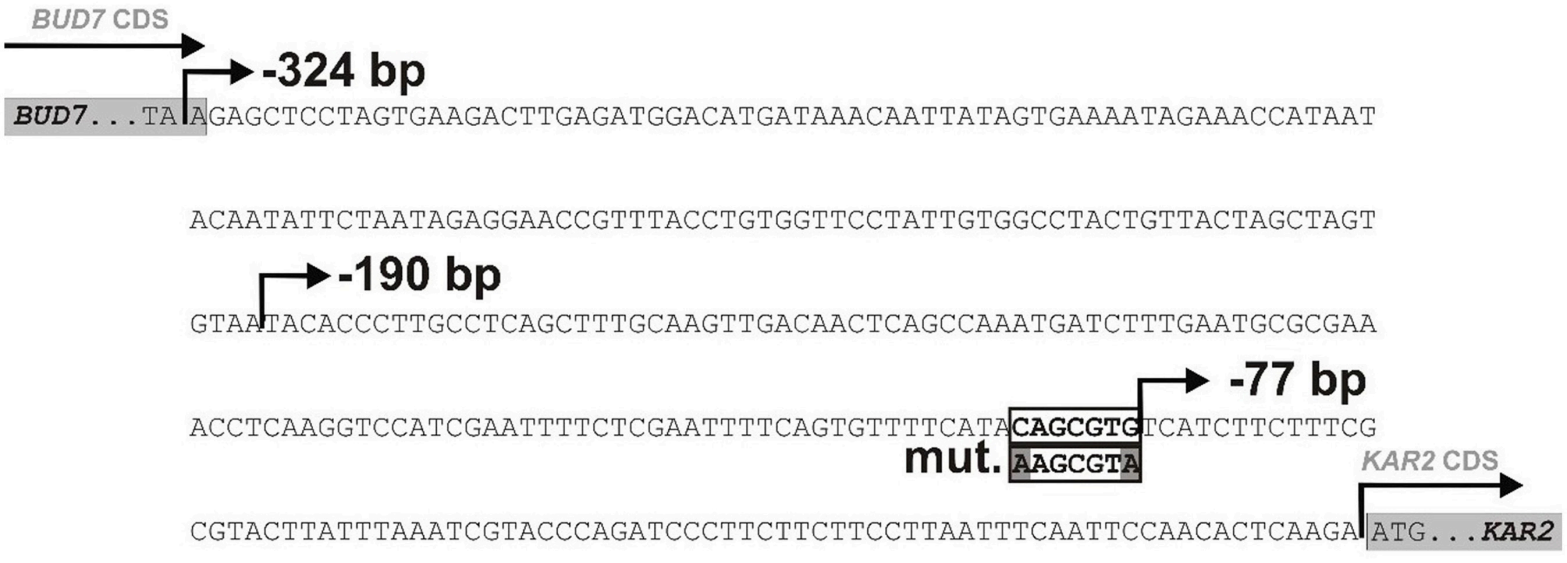
The upstream region of the *KAR2* CDS in *P. pastoris X33*. The nucleotide sequence located upstream of the *KAR2* CDS and downstream of the CDS of the previous gene (B*UD7*) in the genome of *P. pastoris* X33. One potential UPRE (CAGCGTG) was identified in the *KAR2* upstream region, starting −84 bp upstream of the *KAR2* CDS. The following variants of the *KAR2* upstream region were used to control the expression of *sfGFP*:
full length *KAR2* upstream region (FL, −324 bp);truncated variant of *KAR2* upstream region, still containing native UPRE sequence (−191 bp);truncated variant of *KAR2* upstream region with two single mutations (84C→A, 78G→A) in the UPRE sequence (−191 bp mut.); truncated variant of *KAR2* upstream region lacking the UPRE sequence (−77 bp).

We aimed to use the *KAR2* upstream region for monitoring UPR in *P. pastoris* during bioreactor cultivations. The *KAR2* promoter within the *KAR2* upstream region has not yet been characterized in *P. pastoris*. So far, one copy of a potential UPRE (unfolded protein responsive element) sequence, which is a binding site of the transcription activator Hac1p of UPR-involved genes (Mori et al., 1996, 1998), was revealed in the *KAR2* upstream region of *P. pastoris* (Guerfal et al., 2010). The potential UPRE sequence is CAGCGTG, starting −84 bp upstream to the *KAR2* ATG (Figure 1). We prepared mutated variants (truncation and/or single nucleotide mutations) of the *P. pastoris KAR2* upstream region to check for the UPR-function of the potential UPRE (Figure 1). These variants were namely: (1) full length *KAR2* upstream region starting −324 bp upstream *KAR2* CDS (named FL); (2) truncated variant of the *KAR2* upstream region starting −190 bp upstream *KAR2* CDS, still containing the native UPRE sequence (named −190 bp); (3) truncated variant of the *KAR2* upstream region starting −190 bp upstream *KAR2* CDS and with two single mutations (84C → A, 78G → A) disturbing the UPRE sequence (named −190 bp mut.); (4) truncated variant of the *KAR2* upstream region starting −77 bp upstream *KAR2* CDS, thus lacking the UPRE sequence (named −77 bp). These variants were inserted in front of the sfGFP gene in the pREP-*P*_KAR2_-sfGFP plasmid, so the variant of the *KAR2* upstream region controlled the expression of sfGFP. Plasmid pREP-x-sfGFP, having no promoter in front of the sfGFP gene, was used as a negative control. The pREP-*P*_KAR2_-sfGFP plasmids, as well as the pREP-x-sfGFP control plasmid were stably integrated into the *HIS4* locus of the *P. pastoris* X33 strain.

**Figure 2:**
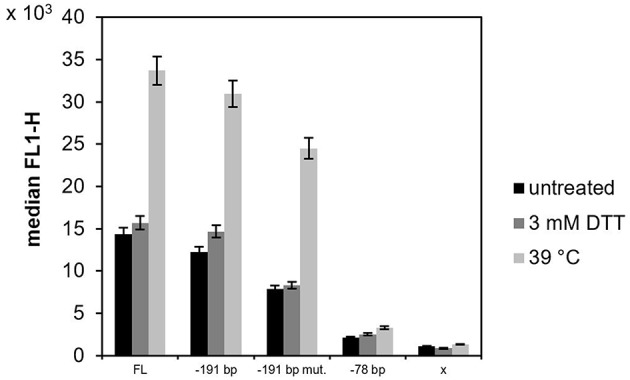
The activity of different variants of the *KAR2* upstream region in *P. pastoris* X33. The promoter activity of the different variants of the *KAR2* upstream region was measured as a green fluorescence of sfGFP (channel FL1) with flow cytometry, as the gene of sfGFP was inserted downstream of the *KAR2* upstream region. A variant with no promoter in front of the sfGFP gene (x) was used as a negative control. *P. pastoris* strains with the different variants of the *KAR2* upstream region (FL, −190 bp, −190 bp mut., −77 bp) controlling *sfGFP* expression, and the negative control strain (*x-sfGFP*) were cultured overnight in shake flasks with YPD, then split into three parallels and further incubated for another 2 h. One parallel was cultured under normal conditions without experimental stress (black bars), the second parallel was cultured in the presence of 3 mM DTT (dark gray bars) and the third parallel was cultured at an increased temperature of 39°C (light gray bars). The displayed values are median FL1 values of all events belonging to a gate defined in a FSC-SSC dot plot (distinguishing *P. pastoris* cells from the background, data not shown). The error bar is showing a standard deviation of three measurements.

The strains were cultured in shake flasks with 100 mL YPD overnight to OD_600_ 2.0–3.0. The culture was then split into three parallels and incubated for 2 h. One of the parallels was cultured under normal (non-stress) conditions, while the other two parallels were cultured under experimentally-induced stress conditions, i.e., either in presence of 3 mM dithiothreitol (DTT) or under increased temperature (39°C). The activity of the different *KAR2* promoter variants was assessed according to the fluorescence of sfGFP with flow cytometry. Under non-stress conditions, the activities of the mutated *KAR2* upstream variants were lower than the activity of the full length (FL) variant (Figure 2), namely 1.2-fold in case of the −190 bp variant, 1.8-fold in case of −190 bp mut. variant and 6.7-fold in case of the −77 bp variant. Because the activity of the variant with the mutated potential UPRE sequence (−190 bp mut. variant) was lower than the activity of its non-mutated counterpart (−190 bp variant) and because the activity dramatically decreased when the potential UPRE sequence was missing in the −77 bp variant, we assume that the CAGCGTG might be the consensus UPRE motif in the *P. pastoris KAR2* upstream region, required for the functionality of the *KAR2* promoter. Against our expectations, up-regulation of the *KAR2* upstream region by DTT treatment was insignificant (Figure 2), even in the case of the FL and −190 bp variants. In contrast to DTT, increased temperature of 39°C led to a strong up-regulation of the promoter activity. Compared to the FL variant, which gave the strongest fluorescence signal at the increased temperature, fluorescence of the −190 bp, −190 bp mut., and −77 bp variants were lower (1.1-fold, 1.4-fold, and 10.2-fold, respectively). However, the increase of fluorescence of the heated (39°C) cells, when compared to the non-treated cells, was the most significant in the case of the −190 bp mut. variant (3.1-fold), while for the FL variant it was 2.3-fold, for the −190 bp variant 2.5-fold and for the −77 bp variant 1.5-fold. Because the activity of the variant with the mutated UPRE (−191 bp mut.) increased even more significantly after heat stress than the activity of the non-mutated variant (−191 bp), we assume that the potential UPRE does not function as a heat shock element. The fluorescence of the negative control strain producing no sfGFP (x), i.e., the background fluorescence signal, was considerably lower in comparison to strains with variants of the *KAR2* upstream region. Due to the highest sfGFP levels of the FL variant, we decided to use the plasmid bearing the *P*_KAR2(FL)_-sfGFP to monitor the UPR in *P. pastoris*. The FSC-FL1 dot plots are provided in Supplementary Figure 2.

### Validation of the *P*_**KAR2**_-Based UPR Reporter in a Strain Over-producing Recombinant Protein in a Bioreactor Fed-Batch Cultivation

**Figure 3:**
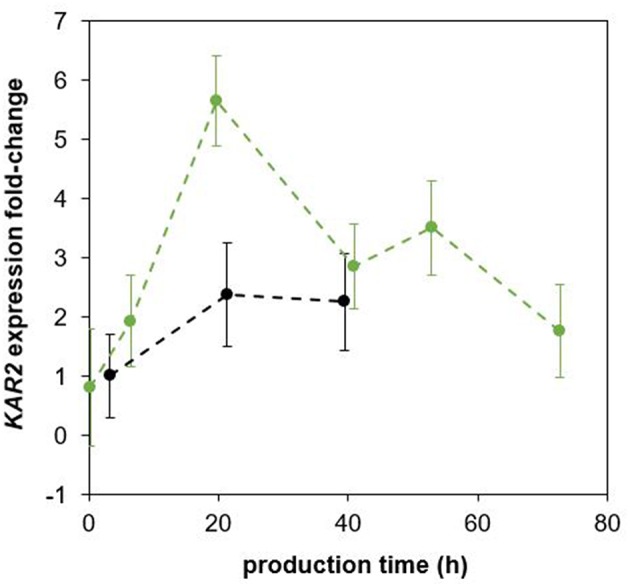
Fold-change of *KAR2* expression in the *P. pastoris* strain producing *Ec*PGA and the non-producing control strain as obtained by qPCR analysis. The *P. pastoris* strains producing *Ec*PGA (green) and the control non-production strain (black) were cultured under the same cultivation conditions (30°C, pH 5.5), maintaining the specific growth rate of biomass with methanol at 0.016 h^−1^. All relative expression data are related to the expression of *KAR2* in the non-producing control strain in the sample taken 3 h after induction.

The plasmid *P*_KAR2(FL)_-sfGFP was integrated into the *P. pastoris* strain producing *Ec*PGA to monitor the UPR during a bioreactor cultivation. Because the *Ec*PGA was previously observed to intracellularly accumulate in *P. pastoris* (Borcinova et al., in preparation; Marešová et al., 2017), we assumed that UPR would be up-regulated, and sfGFP would be strongly produced. The up-regulation of UPR upon production of *Ec*PGA was proved both by flow cytometric analysis of sfGFP fluorescence (Supplementary Figures 4, 5) and qPCR analysis of *KAR2* expression (Figure 3), when comparing the strain producing *Ec*PGA with a control strain producing no recombinant protein, both having the *P*_KAR2(FL)_-sfGFP cassette integrated into their genome.

**Figure 4:**
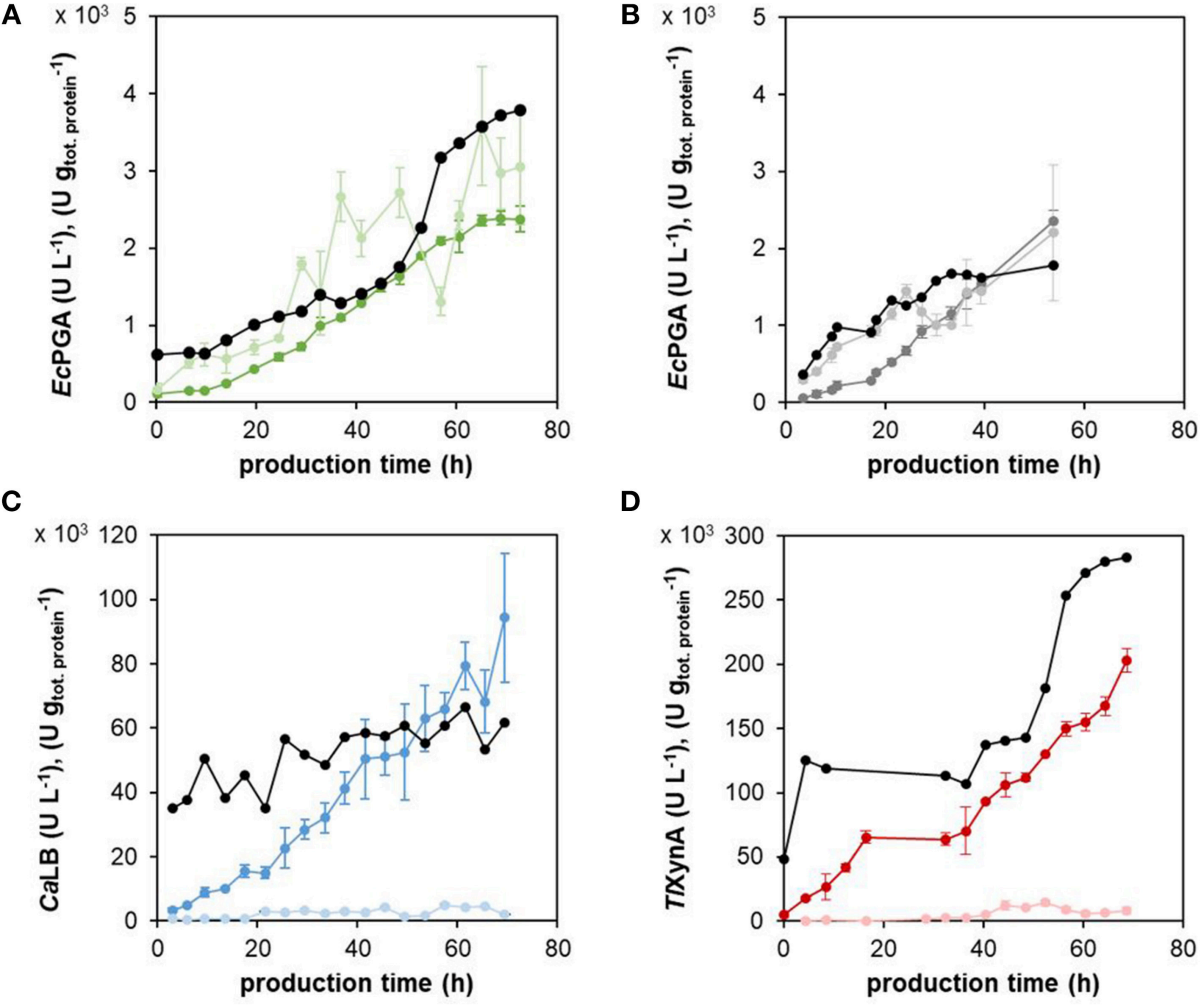
The enzymatic activity of the different recombinant proteins in the centrifuged culture medium and cell extracts. The production of *Ec*PGA in the strain with the expression cassette *P*_KAR2(FL)_-sfGFP **(A)** and in the control strain **(B)**, *Ca*LB **(C)**, and *Tl*XynA **(D)** was assessed as enzymatic activity (U L^−1^) both in the culture medium, i.e., in the supernatant (dark green, dark gray, dark blue, dark red for *Ec*PGA, *Ec*PGA, *Ca*LB, *Tl*XynA, respectively), as well as in the cell extracts, i.e., inside the cells (light green, light gray, light blue, light red for *Ec*PGA, *Ec*PGA, *Ca*LB, *Tl*XynA, respectively). The concentrations were recalculated to the volume of the culture broth. The activities of the extracellular (secreted) enzyme were related to the mass of total extracellular protein (U g${}_{\text{tot}.\text{protein}}^{-1}$, black in **A–D**). The strains with the integrated *P*_KAR2(FL)_-sfGFP cassette for the monitoring of UPR and producing *Ec*PGA **(A)**, *Ca*LB **(C)**, or *Tl*XynA **(D)**, and the control strain with the integrated x-sfGFP cassette producing *Ec*PGA **(B)** were cultured under the same cultivation conditions (30°C, pH 5.5, μ_methanol_ 0.016 h^−1^).

Furthermore, we needed to exclude any potential negative effects of sfGFP production on the strain's growth, physiology and production properties, even though it was shown in *S. cerevisiae* that sfGFP fused to Kar2p did not significantly influence the growth of the strain, nor activated the UPR (Lajoie et al., 2012). Therefore, we prepared a control strain by integrating the negative control cassette x*-*sfGFP (lacking a promoter upstream the *sfGFP*, so producing no sfGFP, Figure 2), instead of the UPR reporter cassette (*P*_KAR2(FL)_-sfGFP), to the *Ec*PGA-producing strain. The strains were cultured under the same cultivation conditions in bioreactors (Table 1). The specific growth rate with methanol during the production phase (Table 1), the production of *Ec*PGA (Figure 4) and viability (Supplementary Figure 6) were comparable in the two *Ec*PGA-producing strains, suggesting that the production of sfGFP did not impair the growth, production properties, nor viability of the *Ec*PGA-producing strain.

Since the production of sfGFP was shown not to influence the strain's physiology and turned out to be detectable with flow cytometry upon the up-regulation of the UPR, we decided to use the *P*_KAR2(FL)_-sfGFP reporter system to monitor the UPR in two other recombinant strains, producing *Ca*LB and *Tl*XynA.

### Evaluation of the Flow Cytometry Data From Bioreactor Cultivations

The *P. pastoris* strains producing and secreting *Ec*PGA, *Ca*LB and *Tl*XynA with the integrated *P*_KAR2(FL)_-sfGFP cassette, the control strain with the integrated *P*_KAR2(FL)_-sfGFP cassette producing none of the recombinant proteins, as well as the control strain without the integrated control x-sfGFP cassette producing *Ec*PGA were cultured under the same cultivation conditions (30°C, pH 5.5) during fed-batch cultivations with methanol, maintaining μ_methanol_ at 0.016 h^−1^ (ca 30% of μ_max, methanol_ of the strains). The achieved specific growth rate with methanol of all the strains was comparable (Table 1).

Flow cytometry was used to assess cell size and complexity (FSC and SSC, respectively), green fluorescence of sfGFP (FL1) referring to the activity of the *KAR2* promoter (UPR), and cell viability as red fluorescence of PI bound within cells with compromised cell membranes (FL3) during the cultivation. Two sub-populations with respect to the FSC signal were distinguished and observed in all strains over the production phase with methanol (examples of density plots are shown in Supplementary Figure 3). It is apparent from the FSC-FL1 dot plot overlay (Supplementary Figure 4) that the FL1 signal (fluorescence of sfGFP) correlated with FSC to some extent, and that the increase in FL1 over time was more significant in the case of cells with higher FSC. The time course of sfGFP fluorescence (median FL1) evaluated separately for the two sub-populations with different sizes (FSC) for all strains is provided in Supplementary Figure 5.

**Figure 5:**
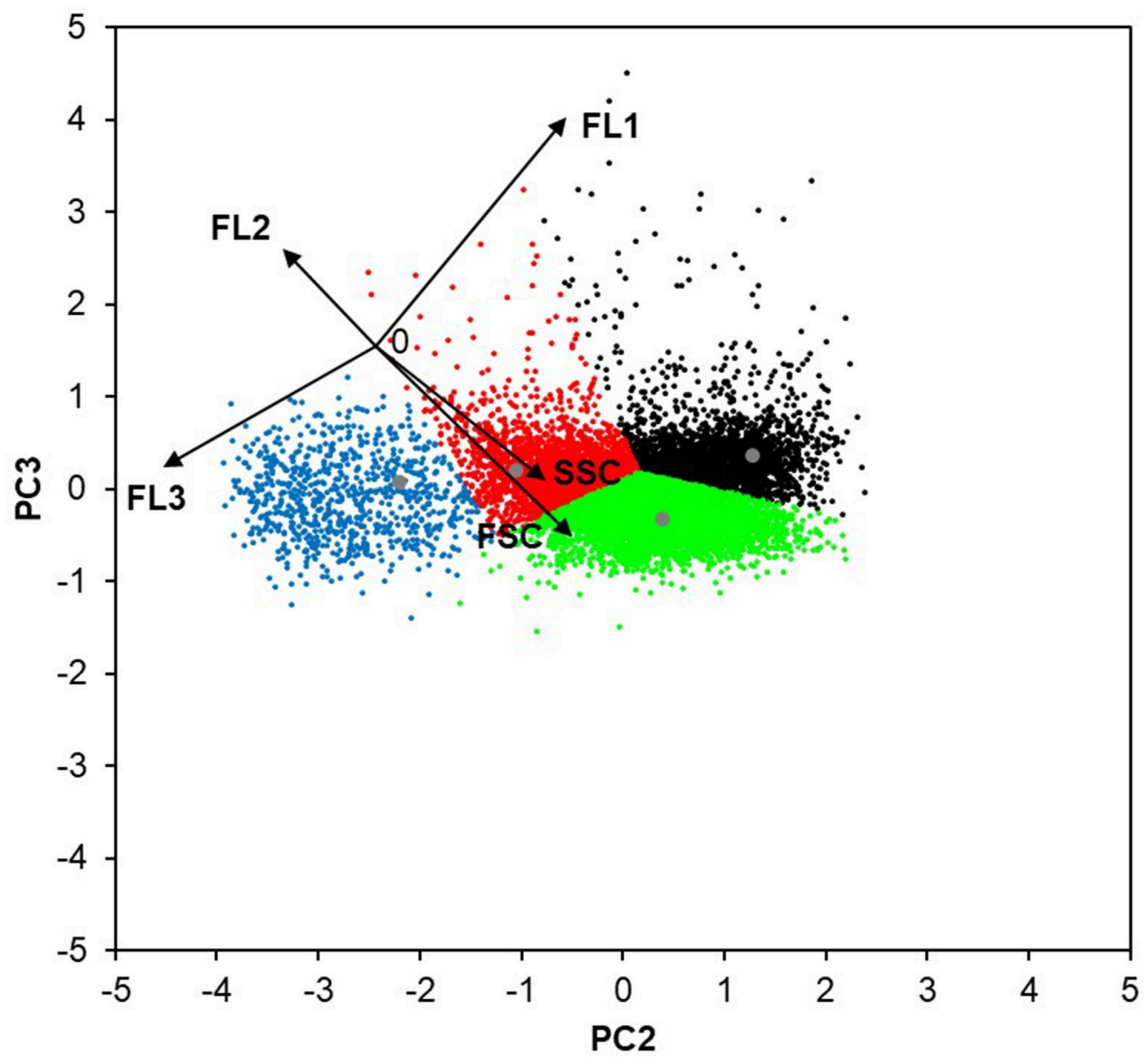
The four sub-populations of *P. pastoris* cells defined with the use of PCA. The flow cytometric data (FSC, SSC, FL1, FL2, FL3) of 10,000 random cells from the bioreactor cultivations of the strains producing *Ec*PGA and *Ca*LB, and the non-producing strain (all at μ_methanol_ 0.016 h^−1^) were analyzed with PCA. The lowest level of redundancy was observed when the data were displayed as the combination of PC2 and PC3 (describing ~39.8% of the population variability), since the flow cytometric data (black vectors) were the most de-correlated. The length of each vector is 50 AU. The combination of PC3 and PC2 was used to establish the clustering method (population centers are displayed in gray). According to the FC signals (black arrows), the identified four clusters can be described as follows: smaller and less complex viable cells with no UPR up-regulation (red); larger and more complex viable cells with no UPR up-regulation (green); viable cells with up-regulated UPR (black); and cells with impaired viability (blue).

In order to reduce redundancy, such as for example the effect of cell size (FSC) on the sfGFP content (FL1) (see Supplementary Figure 4), the flow cytometric data were further evaluated using PCA (Figure 5). Based on the PCA, the following four sub-populations were distinguished: (I) smaller and less complex (lower FSC and SSC) viable cells with no UPR up-regulation; (II) larger and more complex (higher FSC and SSC, also including budding cells, i.e., two incompletely separated cells) viable cells with no UPR up-regulation; (III) viable cells with up-regulated UPR (increased FL1); and (IV) cells with impaired viability (increased FL3).

In order to additionally support the flow cytometry-based results indicating the heterogeneity of *P. pastoris* population with respect to cell size/complexity (FSC, SSC) and green fluorescence (FL1), we performed a shake flask cultivation of the strain producing *Ec*PGA (Pp4) for a microscopic evaluation of the population (Supplementary Figure 7). The strain was cultured in 25 mL BMG with subsequent methanol pulses, and analyzed by flow cytometry and fluorescent microscopy. According to the flow cytometric data (FSC-SSC, FSC-FL1), the division of the population into two sub-populations of different sizes and complexities was less pronounced in a shake flask (Supplementary Figure 7) than in a bioreactor (Supplementary Figure 3). Nevertheless, the population heterogeneity was observed microscopically in the shake flask culture; there were budding cells (two, rarely three connected cells), small oval cells (likely freshly separated cells after division) with weak fluorescence and big round cells with fluorescence of different intensities.

### Physiology- and UPR-Related Effects of Production of Different Heterologous Proteins

The concentration (U L^−1^) of the active enzyme (*Ec*PGA, *Ca*LB and *Tl*XynA) was assessed during fed-batch cultivation (cultivation conditions specified in 3.3) in the centrifuged cultivation medium as well as in the cells (Figure 4). A strong intracellular accumulation of *Ec*PGA was observed, as 50–70% of the total active *Ec*PGA remained in the cells over the whole production phase. In contrast, only a small proportion (2–10%) of the total active *Ca*LB or *Tl*XynA was detected in the cells after 20 h of production. Also, the total protein concentration (g L^−1^) was assessed in the centrifuged cultivation medium and recalculated to the volume of the culture broth (Supplementary Figure 8). The activities of the extracellular (secreted) enzymes related to the mass of total extracellular protein are shown in Figure 4. In case of *Ca*LB (Figure 4C), the amount of active enzyme per mass of total protein (U g${}_{\text{tot}.\text{protein}}^{-1}$) was more or less stable during the production phase, indicating that the portion of the active *Ca*LB in the secreted protein content was constant. In case of *Ec*PGA (Figures 4A,B) and *Tl*XynA (Figure 4D), the increase of active enzyme per mass of total protein after 40–50 h of production indicated an increased portion of the active enzyme in the extracellular protein content.

**Figure 6:**
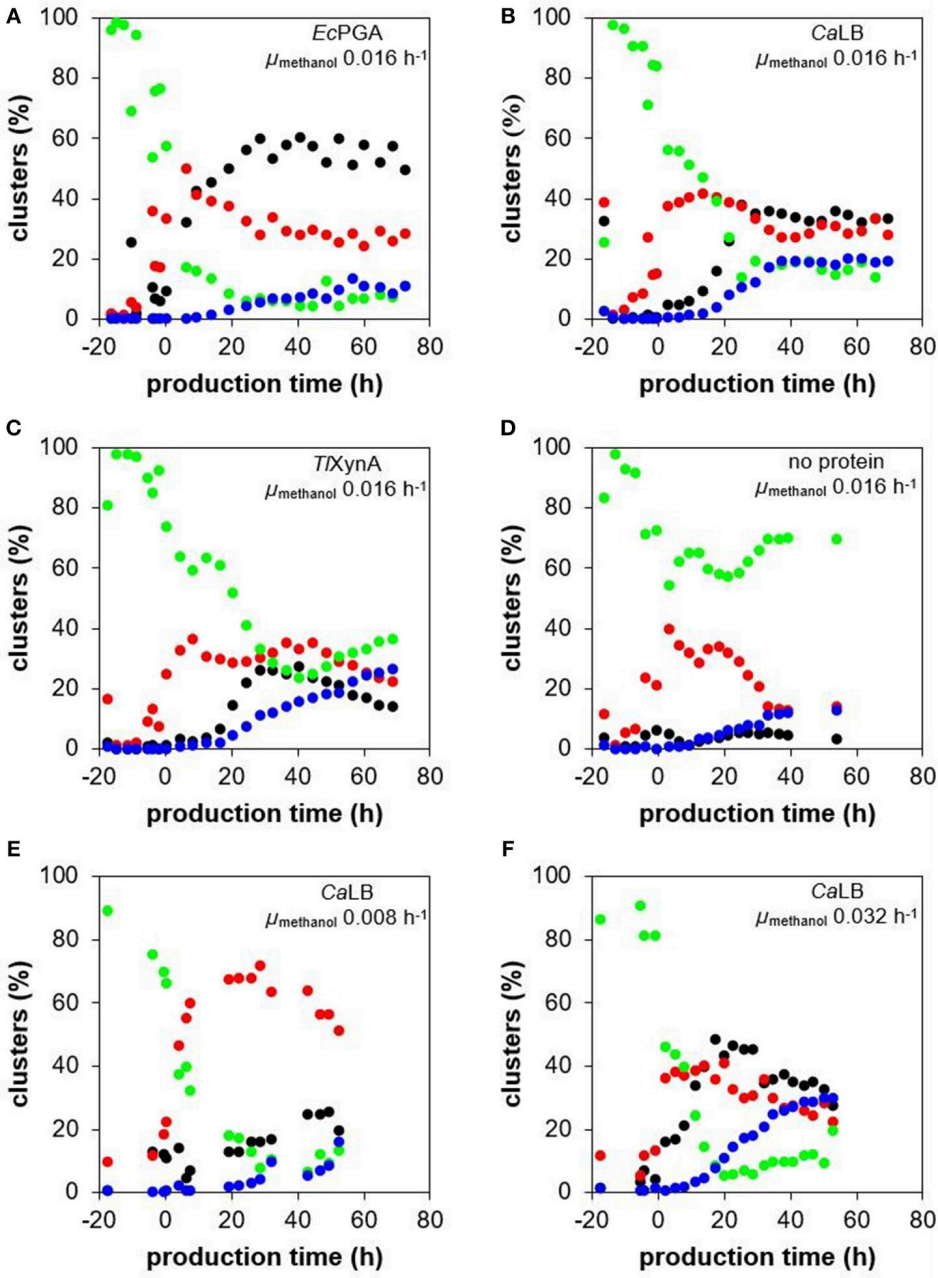
Change of cell size and complexity, UPR up-regulation and viability during cultivations of *P. pastoris* strains producing different recombinant proteins. The *P. pastoris* strains producing *Ec*PGA **(A)**, *Ca*LB **(B)**, or *Tl*XynA **(C)**, as well as a control non-production strain **(D)** were cultured under the same cultivation conditions (30°C, pH 5.5), maintaining the specific growth rate of biomass with methanol at 0.016 h^−1^. Additionally, the *P. pastoris* strain producing *Ca*LB was cultured at 30°C, pH 5.5 and at a specific growth rate of biomass with methanol of 0.008 h^−1^ **(E)** and 0.032 h^−1^ **(F)**. The four sub-populations identified with the PCA of the flow cytometric data were observed in all the cultivation processes: smaller and less complex viable cells with no UPR up-regulation (red ovals); larger and more complex viable cells with no UPR up-regulation (green ovals); viable cells with up-regulated UPR (black ovals); and cells with an impaired viability (blue ovals).

According to the flow cytometric data evaluated with PCA, the cell population was heterogeneous and the distribution of the cells into the four sub-populations (defined in chapter Evaluation of the Flow Cytometry Data From Bioreactor Cultivations) changed over the time course of the cultivation process (Figure 6). In the strains producing different recombinant proteins at the specific growth rate with methanol 0.016 h^−1^ (Figures 6A–C), the amount of the viable smaller and less complex cells increased after the shift to methanol, i.e., the start of the production phase, and was around 30% over the whole production phase. At the same time, the number of the viable bigger cells decreased. In the control non-producing strain (Figure 6D), the number of these smaller and less complex cells began to decrease again after some 20 h of growth on methanol, while the number of the viable larger cells increased proportionally. A decrease in viability was observed in all strains, including the control strain. Around 12% of the population of the control strain was stained with PI at the end of the cultivation, i.e., after 54 h of growth on methanol (Figure 6D), indicating that viability was impaired by long exposure of the cells to methanol. The production of *Ec*PGA did not have any negative effect on cell viability (Figure 6A), since the number of the PI-stained cells was comparable to the control non-producing strain. However, the production of *Ca*LB and *Tl*XynA impaired cell viability compared to the control strain; around 20% of the cells of the *Ca*LB- (Figure 6B) and the *Tl*XynA-producing strains (Figure 6C) had damaged membranes after 54 h of production. The number of damaged cells within the *Tl*XynA-producing strain further increased and was 27% by the end of the process, i.e., after 69 h of production (Figure 6C). The raw flow cytometry data showing the percentage of non-viable (PI-stained) cells are shown in Supplementary Figure 6. It can be seen that in case of *Tl*XynA, the percentage of the cells with impaired viability differed significantly in the two sub-populations of different FSC. While the viability of the “smaller” cells was comparable to the control strain (Supplementary Figure 6A), the viability of the “bigger” cells decreased dramatically (Supplementary Figure 6B).

There was only a slight up-regulation of UPR (around 4% of the population) observed in the non-producing control strain (Figure 6D). In contrast, a significant up-regulation of UPR was observed in all the production strains (Figures 6A–C). In the strain producing and also considerably intracellularly accumulating *Ec*PGA (Figure 4A), the number of cells with up-regulated UPR began to increase steeply right after induction of production (Figure 6A). After ~30 h of production and until the end of the process, up to 60% of the cell population had an up-regulated UPR. Interestingly, in the strains producing *Ca*LB (Figure 6B) and *Tl*XynA (Figure 6C), the maximum portion of cells with up-regulated UPR (35% in case of *Ca*LB production and 26% in case of *Tl*XynA production) was also reached after 30 h of production, although further development was different. The portion of cells with up-regulated UPR was stable over the whole production phase in the *Ca*LB-producing strain, while it began to decrease in the *Tl*XynA-producing strain after 40 h of production and was 14% at the end of cultivation. The stagnation/decrease in the number of cells with an up-regulated UPR might have been caused by lysis of the damaged cells, which was enhanced in the case of production of *Ca*LB and *Tl*XynA, and a consequent release of the intracellular contents, including sfGFP, to the cultivation medium. Alternatively, also a potential loss of the expression cassette (Zhu et al., 2009) might have contributed to the stress relief, i.e., stagnation of the portion of cells with up-regulated UPR.

**Figure 7:**
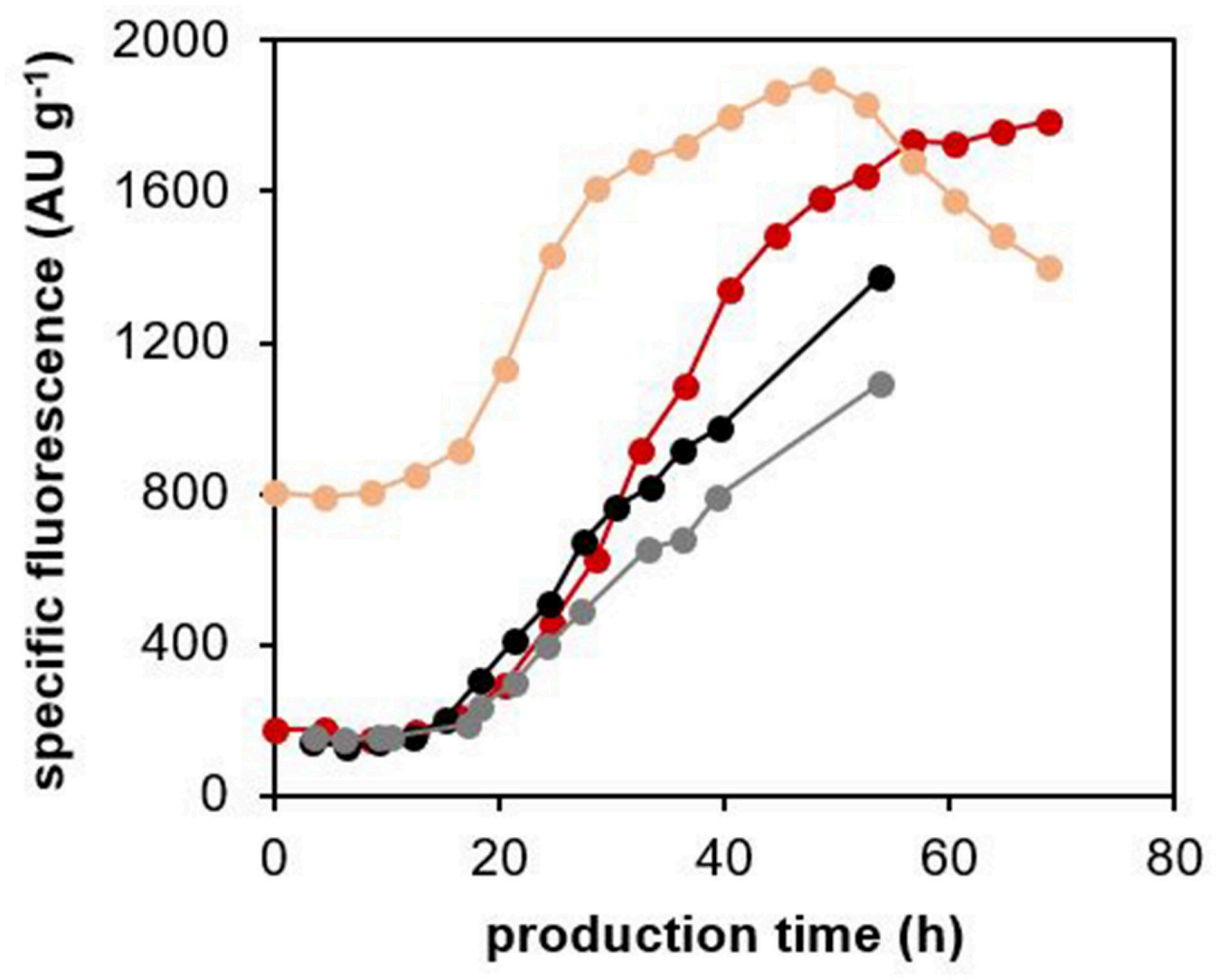
Specific fluorescence of the culture medium and cell lysates of the *P. pastoris* strain producing *Tl*XynA, and the control strains. The *P. pastoris* strain producing *Tl*XynA was cultured at 30°C and pH 5.5, maintaining the specific growth rate of biomass with methanol during the production phase at 0.016 h^−1^. Fluorescence of the centrifuged culture medium and of the cell lysates were measured with a fluorimeter. Specific extracellular fluorescence (dark red) and specific intracellular fluorescence (light red) were calculated from the measured fluorescence (AU) and CDW (g) values. As controls, the specific extracellular fluorescence of the *Ec*PGA-producing strain with no sfGFP production (gray), and the non-producing strain (black) are shown. The control strains were cultured under the same cultivation conditions as the *Tl*XynA-producing strain.

We further investigated the potential leakage of sfGFP from the cells by measuring the fluorescence of the cultivation medium (extracellular fluorescence) with a microplate fluorimeter. We compared the specific extracellular fluorescence (AU gCDW^−1^) of the cultivation medium from the processes with the *Tl*XynA-producing strain, the non-producing control strain, and the control strain producing *Ec*PGA, but no sfGFP (Figure 7). For the *Tl*XynA-producing strain, we also measured the fluorescence of the cell lysate (intracellular fluorescence). The specific extracellular fluorescence (AU gCDW^−1^) in the case of the *Tl*XynA-producing strain began to increase after 15 h of the production phase, and continued increasing until the end of the cultivation. An increase in specific extracellular fluorescence was also observed for the control strains. Therefore, we assume that the increase in extracellular fluorescence was partly caused by the fluorescent background of the culture medium fed into the bioreactor. Nevertheless, after 30 h of production, the specific extracellular fluorescence in the cultivation with the *Tl*XynA-producing strain started to be higher than the specific extracellular fluorescence in cultivations with the control strains. Around the same time point, the number of cells with up-regulated UPR started to decrease, and the number of PI-stained cells in the population exceeded 12% (Figure 6C). The specific intracellular fluorescence measured with the fluorimeter also started to increase after the first 13 h of production, increased steeply until 30 h of production, and then less steeply until 50 h of production. After 50 h of production, it began to decrease. Based on these results, we assume that some of the *Tl*XynA-producing cells were really lysed and releasing sfGFP to the medium. The extracellular sfGFP is not measurable with flow cytometry, which can only detect fluorescence of the cells. Therefore, with our method, we were not able to detect cells with up-regulated UPR and damaged membranes, as a separate cluster.

### The Influence of Specific Growth Rate on *Ca*LB Production, Physiology and ER-Stress

The *P. pastoris* strain producing and secreting *Ca*LB was cultured at 30°C and pH 5.5 during fed-batch cultivations with methanol, maintaining the specific growth rate of biomass with methanol at 0.008 h^−1^, 0.016 h^−1^ or 0.032 h^−1^ (Table 1), which corresponded to 15, 30, and 60% of the maximum specific growth rate with methanol, respectively.

**Figure 8:**
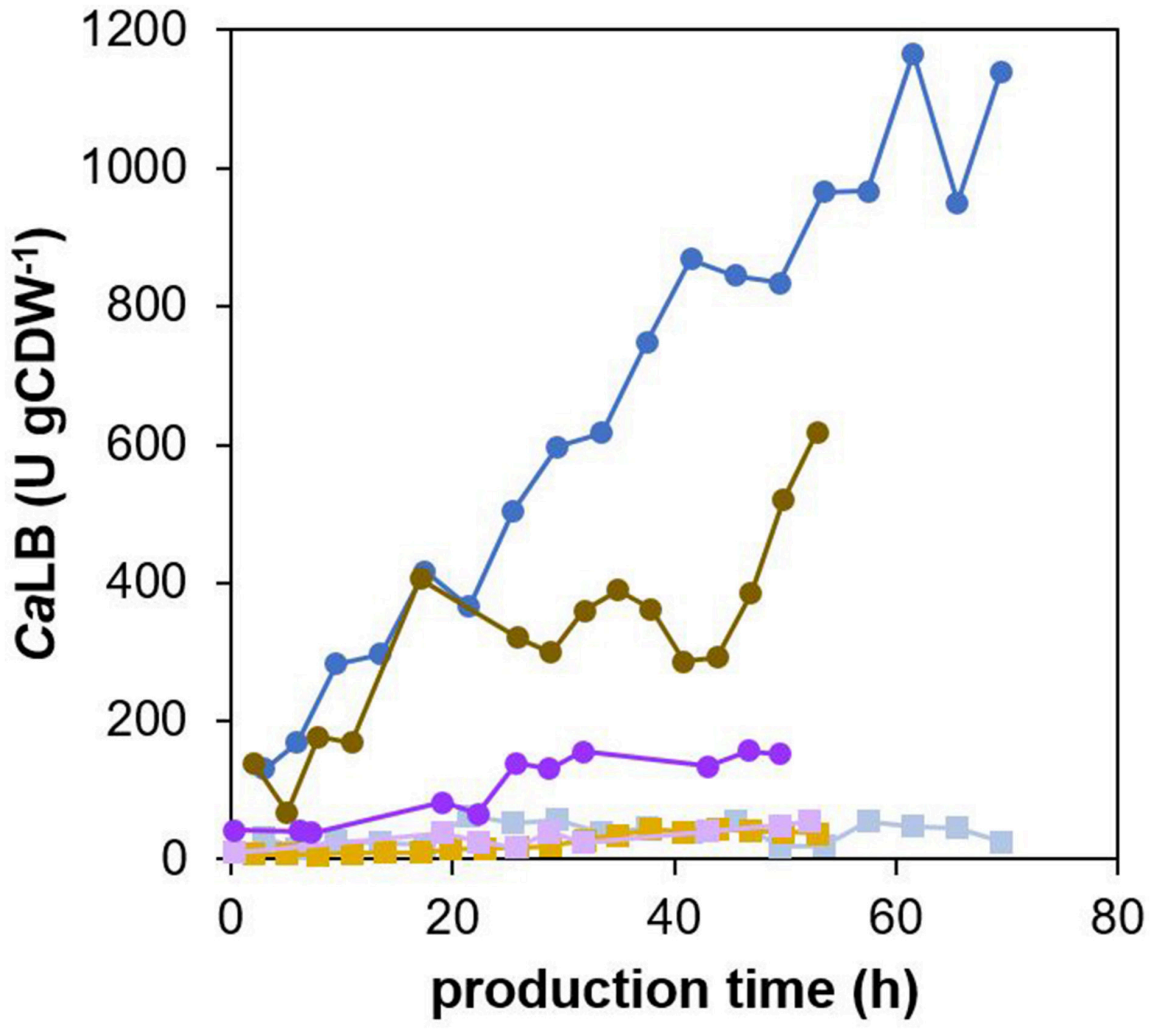
*Ca*LB/biomass yield during cultivations of *P. pastoris* producing recombinant *Ca*LB under different specific growth rates with methanol. The *P. pastoris* strain producing *Ca*LB was cultured at 30°C and pH 5.5, maintaining the specific growth rate of biomass with methanol during the production phase at either 0.008 h^−1^ (purple), 0.016 h^−1^ (blue), or 0.032 h^−1^ (brown). The secreted (dark purple/blue/brown circles) as well as the intracellular (light purple/blue/brown squares) *Ca*LB/biomass yield were calculated.

The concentration of the product was assessed in the culture broth as well as in the cell extracts, and the specific yield of *Ca*LB was calculated (Figure 8). During any of the cultivations with the three different specific growth rates with methanol, the product did not accumulate significantly inside the cells. During cultivation with a specific growth rate with methanol of 0.016 h^−1^, the yield of secreted *Ca*LB increased steadily and was around 1,200 U gCDW^−1^ at the end of the process, i.e., after 70 h of production. In the cultivation with a specific growth rate with methanol of 0.032 h^−1^, the yield of the secreted *Ca*LB increased less steeply than in the cultivation with μ = 0.016 h^−1^ and was 600 U gCDW^−1^ at the end of the process, i.e., after 53 h of production. The lowest yield of the secreted *Ca*LB, 150 U gCDW^−1^ after 50 h of production, was reached in the cultivation with a specific growth rate with methanol of 0.008 h^−1^.

The distribution of the cell population into four sub-populations, as determined by flow cytometry (PCA), differed for the three different specific growth rates with methanol over the time course of the process (Figure 6). The amount of viable smaller and less complex cells during the production phase was significantly higher (between 50 and 70%) at a growth rate of 0.008 h^−1^ (Figure 6E), than at growth rates of 0.016 h^−1^ (Figure 6B) or 0.032 h^−1^ (Figure 6F), where the portion of the viable smaller and less complex cells was 40% or lower during the production phase. The highest portion of cells with damaged membranes (30% after 53 h of production) was observed at μ 0.032 h^−1^ (Figure 6F); at μ 0.016 h^−1^, it was around 20% after 53 h of production (Figure 6B), and at μ 0.008 h^−1^, it was around 16% after 52 h of production (Figure 6E). The greatest impairment to the viability at μ 0.032 h^−1^ cannot be attributed to a high yield of secreted *Ca*LB (the highest was reached at μ 0.016 h^−1^), nor to the faster feeding of methanol, since the portion of PI-stained cells in a non-producing control strain cultured at μ 0.032 h^−1^ did not exceed 13% within a 55 h production phase (Supplementary Figure 9). The number of cells with up-regulated UPR slightly increased over the whole production phase at μ 0.008 h^−1^ and was 20–25% at the end of the process (Figure 6E). During the process with μ 0.032 h^−1^, the maximum portion of cells with up-regulated UPR (48%) was reached after 17 h of production (Figure 6F), which was more and earlier, compared to cultivation at μ 0.016 h^−1^ (Figure 6B). After 17 h of production, the number of the cells belonging to the cluster with up-regulated UPR began to decrease and was 28% at the end of the process, i.e., after 53 h of production.

## Discussion

### Novel Method of Monitoring Up-Regulation of UPR in *P. pastoris*

In *S. cerevisiae*, the UPRE from the *KAR2* promoter (Cox et al., 1993), or in *Yarrowia lipolytica*, the *KAR2* promoter (Madzak and Beckerich, 2006) were previously used to control the expression of the *lacZ* reporter gene. Induction of the UPR was then monitored as the production of β-galactosidase. We applied for the first time a similar approach to monitor the UPR in the yeast *P. pastoris*. But instead of *lacZ*, we fused the gene of the fast maturating sfGFP (Pédelacq et al., 2006; Khmelinskii et al., 2012) to the *KAR2* promoter (upstream region), to monitor its activity. We showed that the sfGFP signal increased after induction of recombinant protein production (Figure 6; Supplementary Figure 6), which was in accordance with the increase in *KAR2* expression, i.e., the UPR up-regulation (Figure 3). Importantly, the sfGFP fluorescence could have been quickly and easily detected with flow cytometry, which enabled a single-cell, at-line and non-invasive analysis of the UPR during fed-batch bioreactor cultivations. Moreover, cell viability, size and complexity were also analyzed simultaneously with flow cytometry.

### *P. pastoris* Population Is Heterogeneous in Terms of Cell Size and Complexity, Up-Regulation of UPR and Cell Viability During Bioreactor Cultivations

Our work provides an interesting insight into the development of a heterogeneous cell population over the time course of a bioreactor cultivation (Figure 6). After the inoculation of the bioreactor, i.e., at the very beginning of the batch phase, the population was homogeneous, formed by larger and more complex cells (higher FSC). Already during the batch phase and further until the start of the production phase with methanol, the population became heterogeneous in terms of cell size and complexity, as the number of smaller and less complex cells (lower FSC) increased. After the start of the production phase, the increasing number of cells with up-regulated UPR was consistent with the decreasing number of viable large and complex cells, assuming that the UPR was being up-regulated mostly in these larger and complex cells. The number of cells with impaired viability increased with the decreasing number of smaller and less complex cells, as well as with the decreasing number of cells with up-regulated UPR. In other words, damaged cells likely arose from smaller and less complex cells, and partly from the large cells with up-regulated UPR. Also, the number of damaged cells increased at different rates and to different extents, depending on the product and specific growth rate with methanol. Cells with an up-regulated UPR and a damaged membrane were not detectable as a separate cluster with our method; as soon as the membrane was damaged, the sfGFP probably leaked out from the cells, so there was no measurable green fluorescence signal in these damaged cells.

The coexistence of *P. pastoris* cells of different sizes, especially during methanol feeding, was reported previously (Hohenblum et al., 2003). It was also shown that the non-producing cells were larger (as indicated by the mean FSC with flow cytometry) than the cells producing the recombinant antibody Fab fragment (Dragosits et al., 2010). According to another work (Aw et al., 2017), *P. pastoris* strains secreting a higher amount of a recombinant protein were shown to have larger cells (as determined by flow cytometry) than strains producing the same product, but lower titers. In our work, the median FSC during the production phase with methanol was comparable in case of the non-producing control strain (median FSC 14.0) and the strain producing *Ec*PGA (median FSC 15.3). The median FSC of the strains producing *Ca*LB and *Tl*XynA was lower (9.1 and 9.7, respectively). Moreover, in the case of the strain producing *Ca*LB, we observed a correlation between FSC and specific growth rate with methanol. The average FSC value (referring to cell size) over the production phase was 6.6 at the lowest growth rate of 0.008 h^−1^. At growth rate 0.016 h^−1^, the average FSC was 9.1 and at the highest growth rate 0.032 h^−1^, the average FSC was 12.6. This trend was, however, not observed in the control strain producing no recombinant protein; the cell sizes were comparable at the specific growth rates with methanol of 0.016 h^−1^ (FSC 14.0) and 0.032 h^−1^ (FSC 12.1). In *S. cerevisiae*, several studies [summarized by Kacmar et al. (2006)] showed that cell size increased with an increasing specific growth rate. However, the increase in cell size was shown to be caused by the production of ethanol at higher growth rates, rather than by a higher specific growth rate (Kacmar et al., 2006).

The heterogeneity in terms of viability of the *P. pastoris* population producing a recombinant protein has been reported in many works (Hohenblum et al., 2003; Jahic et al., 2003; Hyka et al., 2010; Rebnegger et al., 2014, 2016; Zhong et al., 2014; Wang et al., 2016; Aw et al., 2017; Madjid Ansari et al., 2017; Reséndiz-Cardiel et al., 2017). To our best knowledge, the UPR has not been analyzed at the single-cell level in *P. pastoris* until now.

In our work, we did not measure the secretion and intracellular accumulation of the recombinant proteins (*Ec*PGA, *Ca*LB, *Tl*XynA) at a single-cell resolution, as previously shown using microengraving (Love et al., 2010, 2012) or tagging the protein of interest with eGFP (Broger et al., 2011). It was reported that the population of *P. pastoris* cells constitutively producing different recombinant proteins (eGFP, glycosylated/aglycosylated human Fc fragment) was heterogeneous in terms of protein secretion and growth rate. Independently from the type of recombinant protein, three sub-populations with regard to protein secretion were identified: a large subpopulation (35%) that did not secrete the protein significantly but was viable, a subpopulation secreting the protein consistently, and a subpopulation with a changeable rate of protein secretion (Love et al., 2010, 2012). Heterogeneity in terms of protein production was also reported in the case of *P. pastoris* producing human membrane protein tagged with eGFP (Broger et al., 2011). The co-existence of the sub-populations with different fluorescence intensities indicated varying protein productivity, which was likely caused by varying copy numbers of the recombinant gene in the cells (Cregg et al., 2000). Also non-producing cells were identified within the population (Broger et al., 2011). Combining our method for the single-cell detection of cell size/complexity, UPR and viability with single-cell detection of the recombinant protein, or sorting the cells by FC according to their properties and subsequently measuring the product in the sorted subpopulations, it would be possible to relate the cell types characterized in this work (smaller vs. larger cells, cells with up-regulated UPR, cells with damaged membranes) to their secretion phenotype.

### Production of Recombinant Proteins in *P. pastoris* Up-Regulates UPR and Impairs Viability

All three recombinant proteins used in this work, i.e., *Ec*PGA, *Ca*LB, and *Tl*XynA, up-regulated the UPR in *P. pastoris*, though to different extent, depending on the type of the recombinant protein (*Ec*PGA, *Ca*LB, *Tl*XynA, no protein), and, in the case of *Ca*LB, on the specific growth rate with methanol (0.008 h^−1^, 0.016 h^−1^, 0.032 h^−1^). Interestingly, the maximum number of cells with an up-regulated UPR was always reached after 30 h of production, independent of the product, but probably depending on the specific growth rate with methanol.

The majority of studies report UPR up-regulation by (over)production of a recombinant protein (Hohenblum et al., 2004; Gasser et al., 2007; Resina et al., 2007; Khatri et al., 2011; Sjöblom et al., 2012; Hesketh et al., 2013; Vogl et al., 2014; Zhong et al., 2014; Wang et al., 2017; Yu et al., 2017). Only the induced production of secretory insulin precursor in *P. pastoris* was reported to be accompanied by a decrease in the amount of Kar2p in the cells upon methanol shift production (Vanz et al., 2014). This phenomenon was, however, recently explained not by a down-regulation of UPR, but by secretion of Kar2p along with the recombinant protein to the medium and/or by an enhanced autophagy of the ER, resulting in an extracellular release of Kar2p (Roth et al., 2018).

Generally, up-regulation of UPR may be more significant in a fed-batch mode than in chemostat cultures (Zahrl et al., 2017). Up-regulation of UPR was shown to correlate with the intracellular accumulation of human trypsinogen (Hohenblum et al., 2004) or interleukin (Zhong et al., 2014). In contrast, in the case of partial intracellular accumulation of the mucin-type protein fused with GFP, the ER folding stress was low; however there was a secretory bottle-neck in the Golgi system (Sjöblom et al., 2012). In another work, the UPR was reported to be up-regulated to a different extent in *P. pastoris* clones producing human serum albumin (HSA), which all contained only one copy of the gene coding for HSA (Aw et al., 2017); there was no clear correlation between up-regulation of the UPR, and product titers, but the ERAD was only up-regulated in clones with high secretion of HSA. The UPR was also shown to be down-regulated at lower specific growth rates (Rebnegger et al., 2014). In our work, the increasing specific growth rate with methanol resulted in a faster and stronger up-regulation of UPR.

In this work, the up-regulation was most significant in the case of *Ec*PGA (60% of the *Ec*PGA-producing cells exhibited up-regulated UPR), which strongly accumulated in the cells (up to 50–70% of the total active *Ec*PGA). As already discussed in the work of Marešová et al. (2017), the reason for poor secretion of *Ec*PGA might be its incorrect maturation in *P. pastoris* cells, which results in ER stress. A co-expression of *E. coli* chaperone genes might improve the secretion of proteins of bacterial origin (Summpunn et al., 2018). The recombinant *Ca*LB and *Tl*XynA in our work did not accumulate significantly inside the cells, yet we observed an up-regulation of the UPR. This was probably caused by the overexpression of the recombinant genes from the *AOX1* promoter, which resulted in a high load of protein in the ER. Since the post-translational modifications of *Tl*XynA involve formation of disulfide bonds (Gruber et al., 1998; Damaso et al., 2003), and in the case of *Ca*LB, also glycosylation (Uppenberg et al., 1994), we believe the capacity of the ER was exceeded. The up-regulation of UPR by the production of recombinant xylanase A in *P. pastoris* was reported previously (Lin et al., 2013); compared to a control non-producing strain, the fold change in *KAR2* expression analyzed by qPCR was ~0.5 in a strain carrying one copy of the xylanase gene, and 1.9 in a strain carrying four copies of the xylanase gene. No ERAD up-regulation was observed, indicating that the xylanase was not degraded (Lin et al., 2013). Detectable amounts of Kar2p were also observed in the *P. pastoris* strain producing *Ca*LB (Samuel et al., 2013), but there was no comparison made to a non-producing strain. Overall, our results indicate that the up-regulation of UPR was not exclusively linked to poor secretion of the recombinant protein. Additionally, there was also no clear correlation between the intensity of UPR caused by the different proteins, and the mass of the secreted total protein (g) (Supplementary Figure 8). There is a general demand for more detailed characterization of the secretory machinery and the UPR in *P. pastoris*, information that is currently still derived from other yeasts (Puxbaum et al., 2015).

The viability of the cells was impaired by the production of *Ca*LB and *Tl*XynA, as the number of PI-stained cells in these strains was higher than in the non-producing control strain. In contrast to *Ca*LB and *Tl*XynA, the production of *Ec*PGA in our work did not enhance cellular damage, compared to the non-producing control strain. The impaired viability of *P. pastoris* resulting from production of recombinant protein is commonly observed, although to different extents, depending on the recombinant protein and cultivation conditions. For example, in the case of the production of recombinant human trypsinogen, 65% of cells were non-viable after ~135 h of production with methanol (Hohenblum et al., 2003). The production of porcine trypsinogen at pH 4 and at a high cell density in fed-batch culture resulted in more than 80% of damaged cells (Hyka et al., 2010). High viability was observed at very low, almost zero, specific growth rates (Rebnegger et al., 2016). In another study, however, there was no clear correlation between specific growth rates, viability and product yield; at the lowest specific growth rate (0.015 h^−1^), the viability was 94%, while at the higher specific growth rates, the number of dead cells was even lower (Rebnegger et al., 2014). In our work, the increasing specific growth rate with methanol resulted in a larger fraction of damaged cells, but the highest specific yield of *Ca*LB was reached at the mid value within the range of the tested specific growth rates with methanol, i.e., at 0.016 h^−1^.

## Conclusions

Production and secretion of three different recombinant proteins (*Ec*PGA, *Ca*LB, *Tl*XynA) by *P. pastoris* was investigated along with up-regulation of the UPR and cell viability, which were assessed at the single-cell level in living cells and at-line with flow cytometry. In all three strains, as well as in a non-producing control strain, which were all cultured under the same cultivation conditions, a heterogeneous population consisting of four subpopulations developed after the induction of protein production. The distribution of the population into four fractions differed over the time course of the cultivation process and depended on the recombinant protein and the specific growth rate. Up-regulation of the UPR was especially strong when the recombinant protein was not properly secreted (*Ec*PGA), but was not linked exclusively to the intracellular accumulation. We observed no correlation between up-regulation of UPR and impaired viability, and also no clear correlation between viability and protein production (*Ca*LB). We believe these results emphasize the importance of a complex and systematic understanding of the biotechnological process, with a single-cell analysis of the production population, in order to enhance productivity of the bioprocess. We developed a reporter system for the UPR, which enables fast and easy monitoring of the UPR at the single-cell level and in a non-invasive manner. This method could be adapted to a broad range of biotechnologically important production hosts and also, with no necessary adaptation, could be used at the screening scale. By understanding the relationship between protein production/secretion and the tuning of the UPR, this monitoring system based on fluorescence measurement might be utilized in a feedback control of a bioprocess.

## Author Contributions

HR designed and conducted the experiments and wrote the manuscript. IZ created the program for the analysis of the flow cytometric data. MB and AW participated in the construction of the recombinant strains. PM participated in performing the bioreactor experiments and analyzing the data from the cultivation processes. DM presented this work at the conference Microbial Stress: from Systems to Molecules and Back in Kinsale, Ireland. ZK designed the expression cassette for monitoring the unfolded protein response and supervised the work. AG, KM, and KK supervised the work.

### Conflict of Interest Statement

The authors declare that the research was conducted in the absence of any commercial or financial relationships that could be construed as a potential conflict of interest.
