# Supplementary material for: Single-Cell Approach to Monitor the Unfolded Protein Response During Biotechnological Processes With Pichia pastoris
Source: Front Microbiol. 2019 Feb 27;10:335. doi: 10.3389/fmicb.2019.00335 (PMC6404689; doi:10.3389/fmicb.2019.00335)
Supplement: Supplementary file 1 [file Data_Sheet_1.pdf]

Supplementary Material

Single-cell approach to monitoring the unfolded protein response during biotechnological processes with *Pichia pastoris*

Hana Raschmanová[*], Iwo Zamora, Martina Borčinová, Patrick Meier, Dominik Mächler, Astrid Weninger, Anton Glieder, Karel Melzoch, Zdeněk Knejzlík, Karin Kovar

*** Correspondence:** Hana Raschmanová: raschmah@vscht.cz

1 Supplementary Figures and Tables

1.1 Supplementary Figures

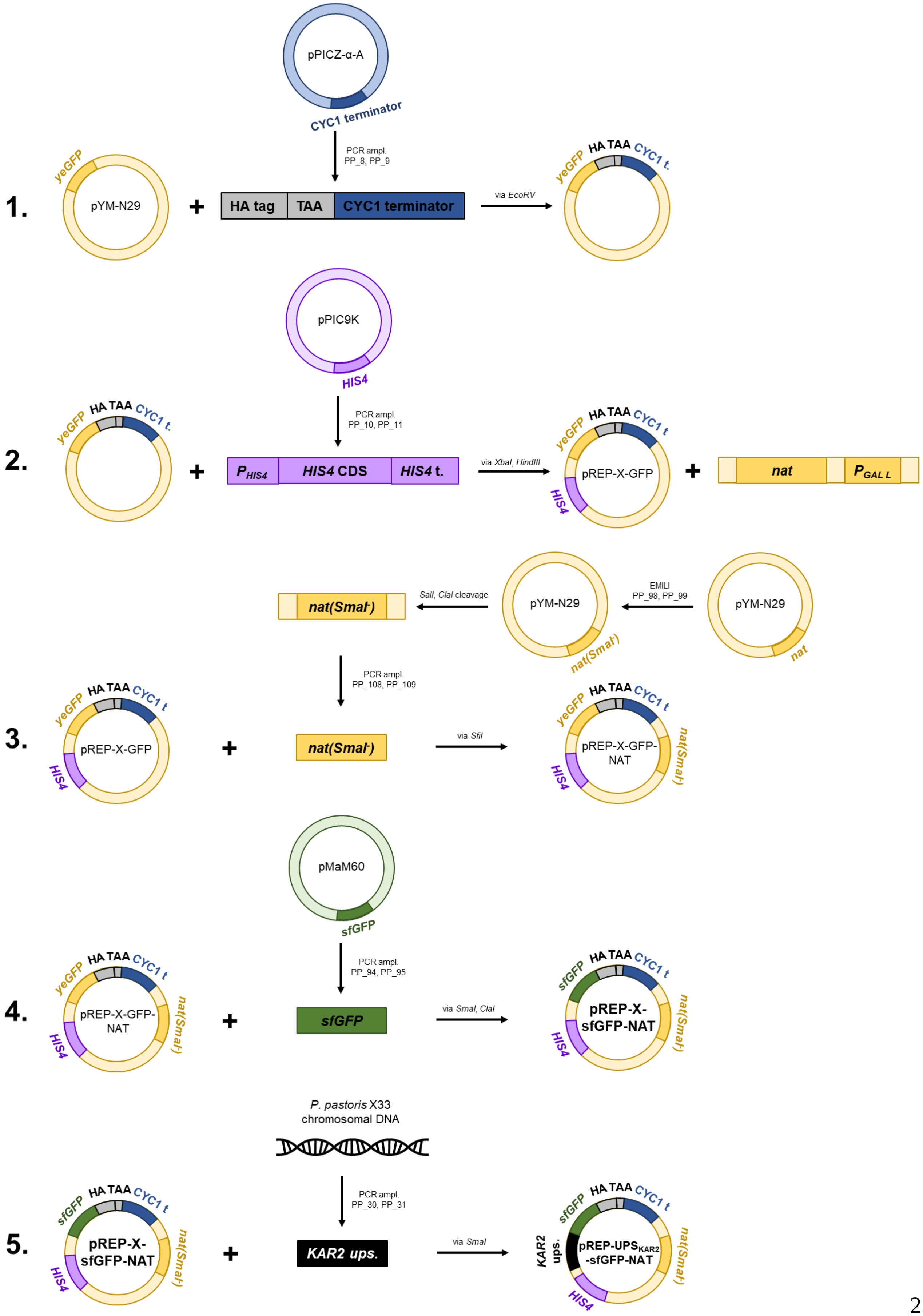
pPICZ-α-A
CYC1 terminator
PCR ampl.
PP_8, PP_9
1.
yeGFP
pYM-N29
+
HA tag
TAA
CYC1 terminator
via EcoRV
yeGFP
HA TAA
CYC1 t.
pPIC9K
HIS4
PCR ampl.
PP_10, PP_11
2.
yeGFP
HA TAA
CYC1 t.
+
P HIS4
HIS4 CDS
HIS4 t.
via XbaI, HindIII
pREP-X-GFP
HIS4
+
nat
P GAL L
nat(SmaI⁻)
SalI, ClaI cleavage
pYM-N29
nat(SmaI⁻)
EMILI
PP_98, PP_99
pYM-N29
nat
PCR ampl.
PP_108, PP_109
3.
pREP-X-GFP
+
nat(SmaI⁻)
via SfiI
pREP-X-GFP-NAT
pMaM60
sfGFP
PCR ampl.
PP_94, PP_95
4.
pREP-X-GFP-NAT
+
sfGFP
via SmaI, ClaI
pREP-X-sfGFP-NAT
P. pastoris X33
chromosomal DNA
PCR ampl.
PP_30, PP_31
5.
pREP-X-sfGFP-NAT
+
KAR2 ups.
via SmaI
pREP-UPS KAR2 -sfGFP-NAT
KAR2 ups.

Supplementary Figure 1. Construction of the plasmid pREP-UPS$_{KAR2}$-sfGFP-NAT.

1. The *CYC1* terminator was PCR-amplified from pPICZ-α-A plasmid (Invitrogen) using primers PP_8 and PP_9. The primer PP_8 also carried the nucleotide sequence of the HA (human influenza hemagglutinin) epitope tag for immunochemical detection, and stop codon TAA. The amplified sequence of the HA epitope tag, stop codon TAA and *CYC1* terminator were inserted downstream of the yeGFP coding sequence in the pYM-N29 vector (EUROSCARF) via restriction site cloning (*EcoRV*).

2. The pYM-N29 bearing the HA epitope and the *CYC1* terminator was then cut by *XbaI* and *HindIII* restriction endonucleases and the *HIS4* cassette, which was PCR-amplified from pPIC9K (Invitrogen) using primers PP_10 and PP_11, was inserted into the backbone. The resulting plasmid was named pREP-X-GFP.

3. Then, a cassette carrying the *nat* gene with a silent mutation was prepared. The GTC codon at 45 nt downstream of ATG in *nat* had to be converted to GTG in order to eliminate the *SmaI* restriction site, as *SmaI* was later used as a cloning site for the introduction of the *KAR2* upstream region into the plasmid. Mutagenesis of the *nat* CDS contained in pYM-N29 (EUROSCARF) was performed using the EMILI method (Fuzik et al., 2014) with primers PP_98 and PP_99. The fragment containing the mutated *nat(SmaI$^-$)* cassette was then removed from plasmid pYM-N29 by the restriction endonucleases *SalI* and *ClaI*, and used as a template for PCR amplification of the mutated *nat(SmaI$^-$)* cassette using primers PP_108 and PP_109. The *nat(SmaI$^-$)* cassette was inserted into the *SfiI* restriction site in the vector pREP-X-GFP. The constructed plasmid was named pREP-X-GFP-NAT.

4. Then, yeGFP was removed from the plasmid pREP-X-GFP-NAT using restriction endonucleases *SmaI* and *ClaI*, and sfGFP was inserted instead. sfGFP was PCR-amplified from pMaM60 (Khmelinskii et al., 2012) using primers PP_94 and PP_95. The constructed plasmid was named pREP-X-sfGFP-NAT.

5. The upstream region of *KAR2* is the nucleotide sequence between the stop codon TAA of the previous gene (*BUD7*) and the start codon ATG of *KAR2*. This sequence was PCR-amplified from *P. pastoris* X33 (Invitrogen) chromosomal DNA using primers PP_30 and PP_31 and inserted into the pREP-X-sfGFP-NAT plasmid via *SmaI* restriction site. The final plasmid was named pREP-UPS$_{KAR2}$-sfGFP-NAT. The nucleotide sequences of all the primers are provided in Supplementary Table 1.

Elements on the plasmids are not drawn to scale.

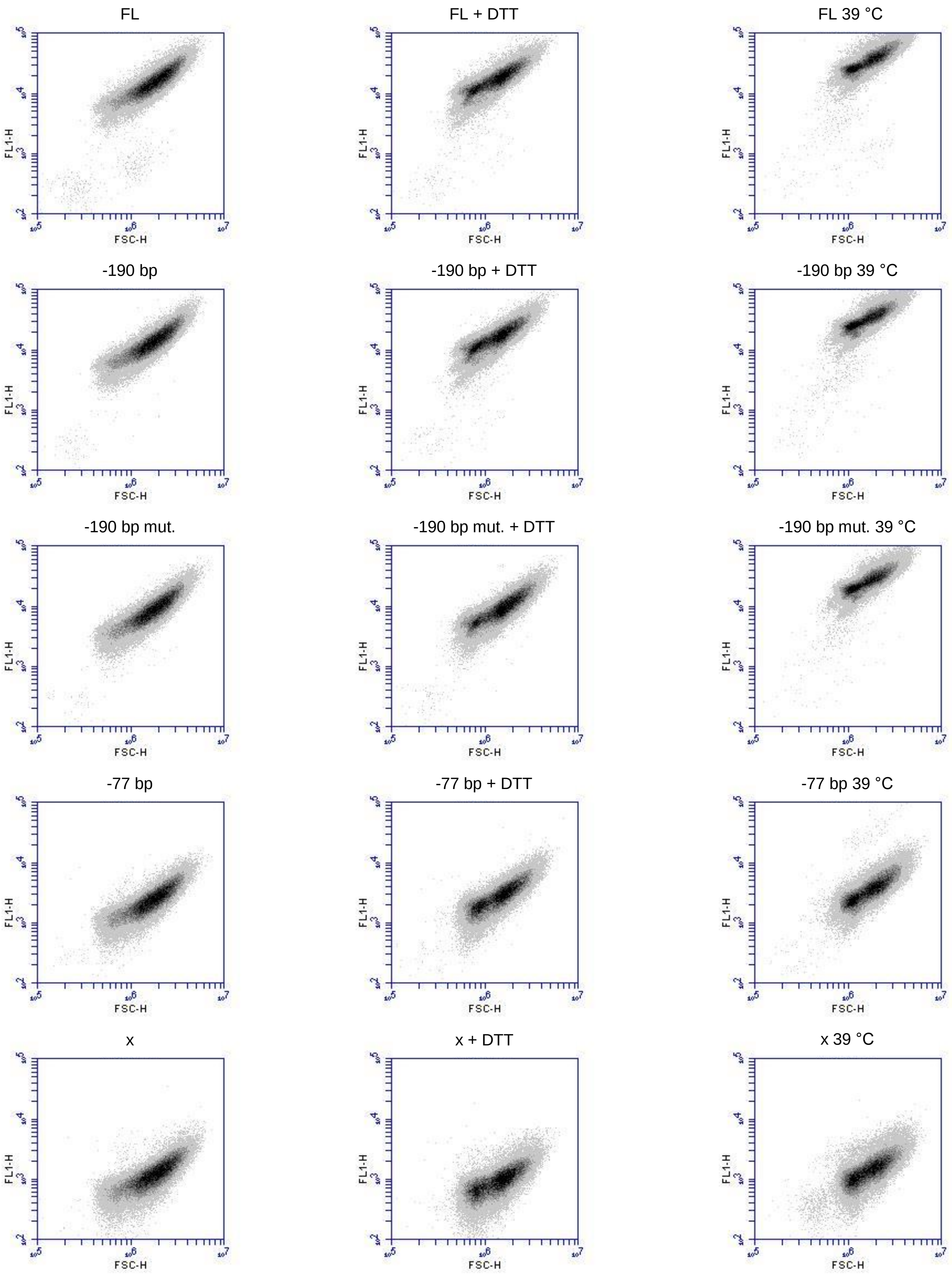
FL
FL + DTT
FL 39 °C
-190 bp
-190 bp + DTT
-190 bp 39 °C
-190 bp mut.
-190 bp mut. + DTT
-190 bp mut. 39 °C
-77 bp
-77 bp + DTT
-77 bp 39 °C
x
x + DTT
x 39 °C
FL1-H
FSC-H

Supplementary Figure 2. FSC-FL1 dot plots of the *P. pastoris* strains with the different variants of the *KAR2* upstream region controlling the expression of sfGFP.

The promoter activity of the different variants of the *KAR2* upstream region was measured as green fluorescence of sfGFP (channel FL1) with flow cytometry (BD Accuri^{TM} C6 flow cytometer), as the gene of sfGFP was inserted downstream of the *KAR2* upstream region. The different variants of the *KAR2* upstream region were: a variant starting 324 bp upstream to the ATG of the *KAR2* cds, named FL (A), a variant starting 190 bp upstream to the ATG of the *KAR2* cds (B), a variant starting 190 bp upstream to the ATG of the *KAR2* cds with a point mutation in the potential UPRE (C), and a variant starting 77 bp upstream to the ATG of the *KAR2* cds, i.e. without the potential UPRE (D). A variant with no promoter in front of the sfGFP gene (x) was used as a negative control (E). The *P. pastoris* strains with different variants of the *KAR2* upstream region (FL, -190 bp, -190 bp mut., -77 bp) controlling *sfGFP* expression, and the negative control strain (x-*sfGFP*) were cultured overnight in shake flasks with YPD, then split into three parallels and further incubated for another two hours. One parallel was cultured under normal conditions without experimental stress (left), the second parallel was cultured in the presence of 3 mM DTT (middle) and the third parallel was cultured at an increased temperature of 39 °C (right). The events displayed in the dot plot belong to a gate defined in a FSC-SSC dot plot (distinguishing *P. pastoris* cells from the background, data not shown).

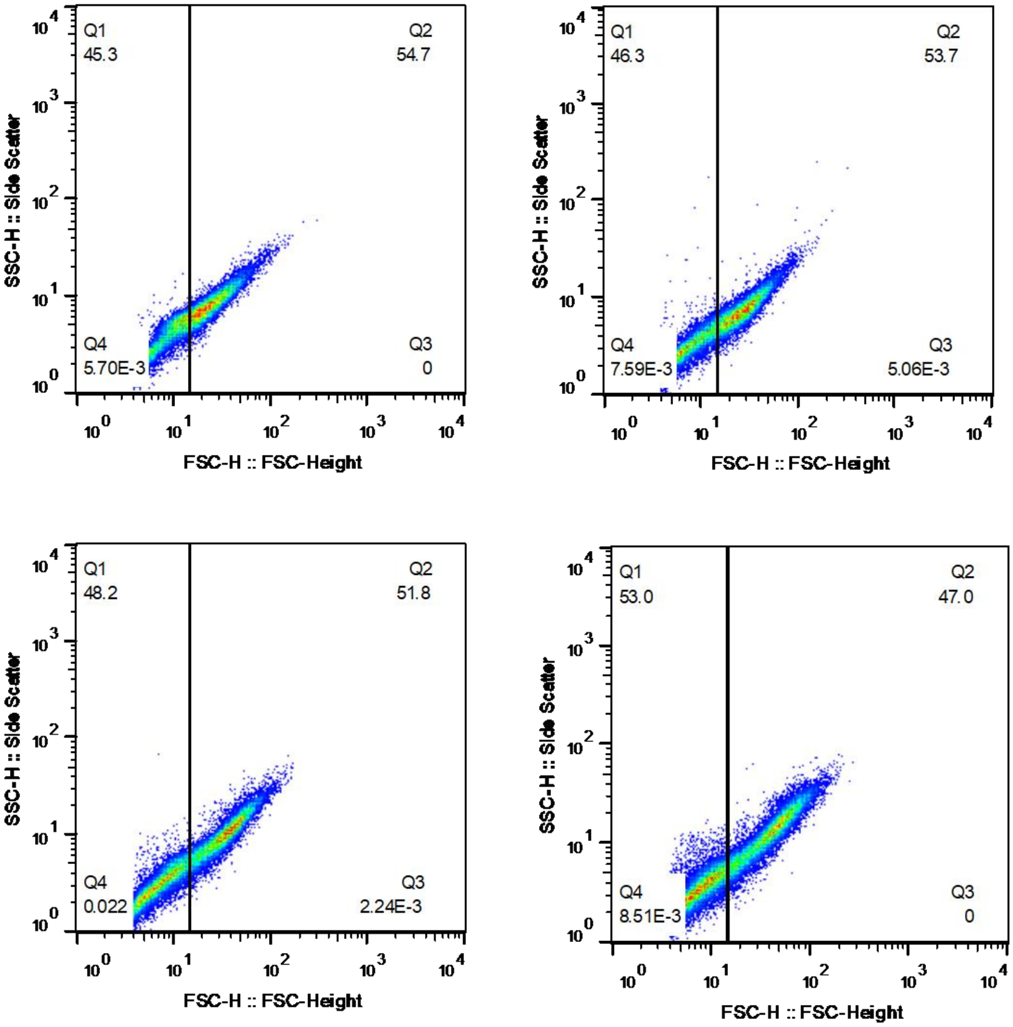

Supplementary Figure 3. FSC-SSC density plot of four samples from bioreactor cultivation of the *P. pastoris* strain producing *Ec*PGA.

The *P. pastoris* strain producing *Ec*PGA was cultured at 30 °C, pH 5.5 and a specific growth rate with methanol of 0.016 h^{-1}. The samples were analyzed by flow cytometry (BD FACSCalibur 4CA), measuring FSC-H and SSC-H (and also FL1-H and FL3-H). In this density plot, the FSC-H and SSC-H values of the samples taken in 0 h (A), 14 h (B), 29 h (C) and 45 h (D) are displayed. The population was divided into two sub-populations, Q1 and Q2, with respect to the FSC-H signal.

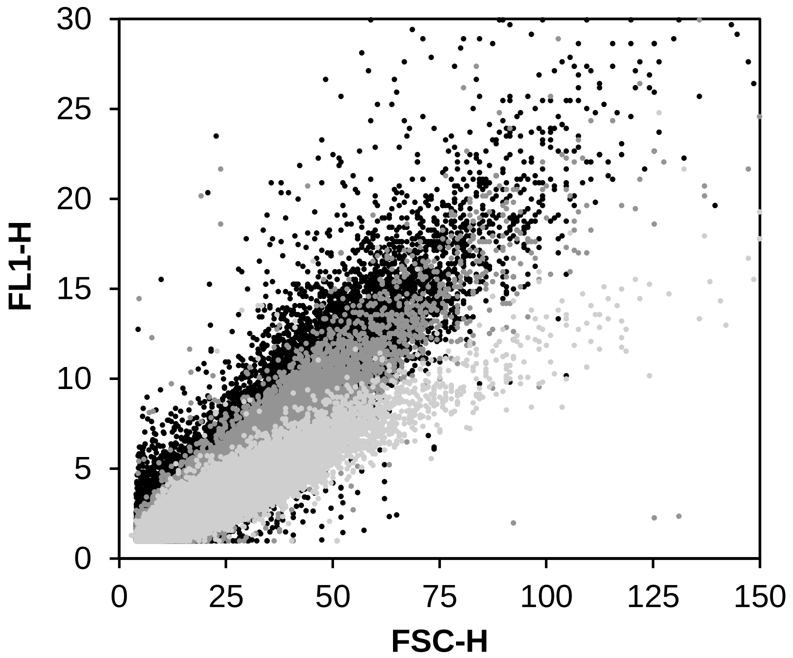

Supplementary Figure 4. FSC-FL1 dot plot of three samples from the bioreactor cultivation of the *P. pastoris* strain producing *Ec*PGA.

The *P. pastoris* strain producing *Ec*PGA was cultured at 30 °C, pH 5.5 and specific growth rate with methanol 0.016 h^{-1}. The samples were analyzed with flow cytometry (BD FACSCalibur 4CA), measuring FSC-H and FL1-H (and also SSC-H and FL3-H). In this dot plot, the FSC-H and FL1-H values of the samples taken in 0 h (light grey), 14 h (dark grey) and 29 h (black) are displayed.

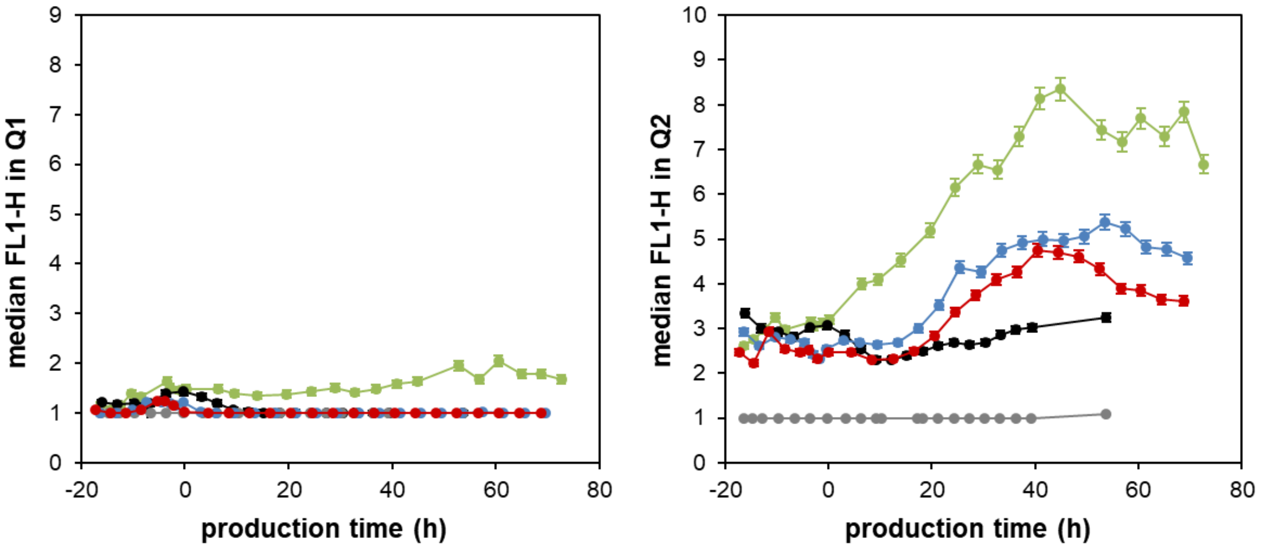

Supplementary Figure 5. Time course of the sfGFP fluorescence signal (detected as median FL1-H) in *P. pastoris* strains cultured in fed-batch cultivations in bioreactors.

The *P. pastoris* strains producing *Ec*PGA (green), *Ca*LB (blue) or *Tl*XynA (red), as well as the control strain producing only *Ec*PGA and not sfGFP (grey), and the control strain producing only sfGFP and no other recombinant protein (black), were cultured at 30 °C, pH 5.5 and a specific growth rate with methanol of 0.016 h^{-1}. The samples were analyzed by flow cytometry (BD FACSCalibur 4CA) and the cell population was divided into two sub-populations with respect to FSC-H: Q1 with lower FSC and Q2 with higher FSC. The fluorescence signal of sfGFP was detected on the FL1 detector and median FL1-H was assessed for the sub-populations Q1 (A) and Q2 (B) separately.

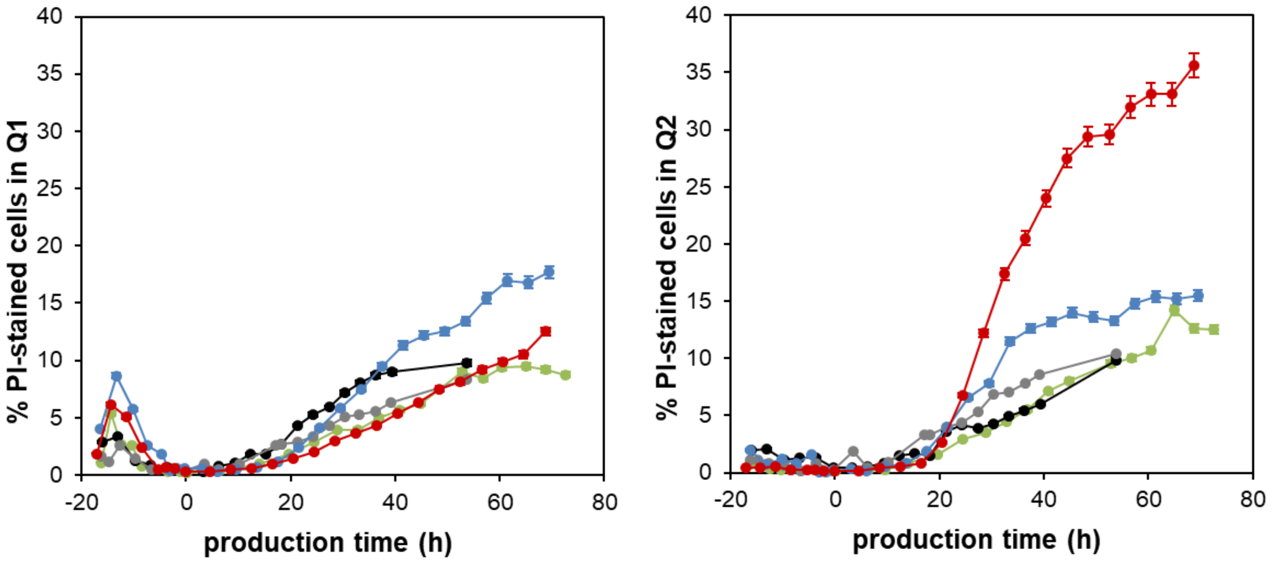

Supplementary Figure 6. Time course of the amount of PI-stained cells (detected as median FL3-H) in *P. pastoris* strains cultured in fed-batch cultivations in bioreactors.

The *P. pastoris* strains producing *Ec*PGA (green), *Ca*LB (blue) or *Tl*XynA (red), as well as the control strain producing only *Ec*PGA and not sfGFP (grey), and the control strain producing only sfGFP and no other recombinant protein (black), were cultured at 30 °C, pH 5.5 and a specific growth rate with methanol of 0.016 h^{-1}. The samples were analyzed by flow cytometry (BD FACSCalibur 4CA) and the cell population was divided into two sub-populations with respect to FSC-H: Q1 with lower FSC and Q2 with higher FSC. The viability of the cells was assessed after staining with propidium iodide (PI). PI was detected on the FL3 detector. The percentage of the PI-stained cells was assessed for the populations Q1 (A) and Q2 (B) separately.

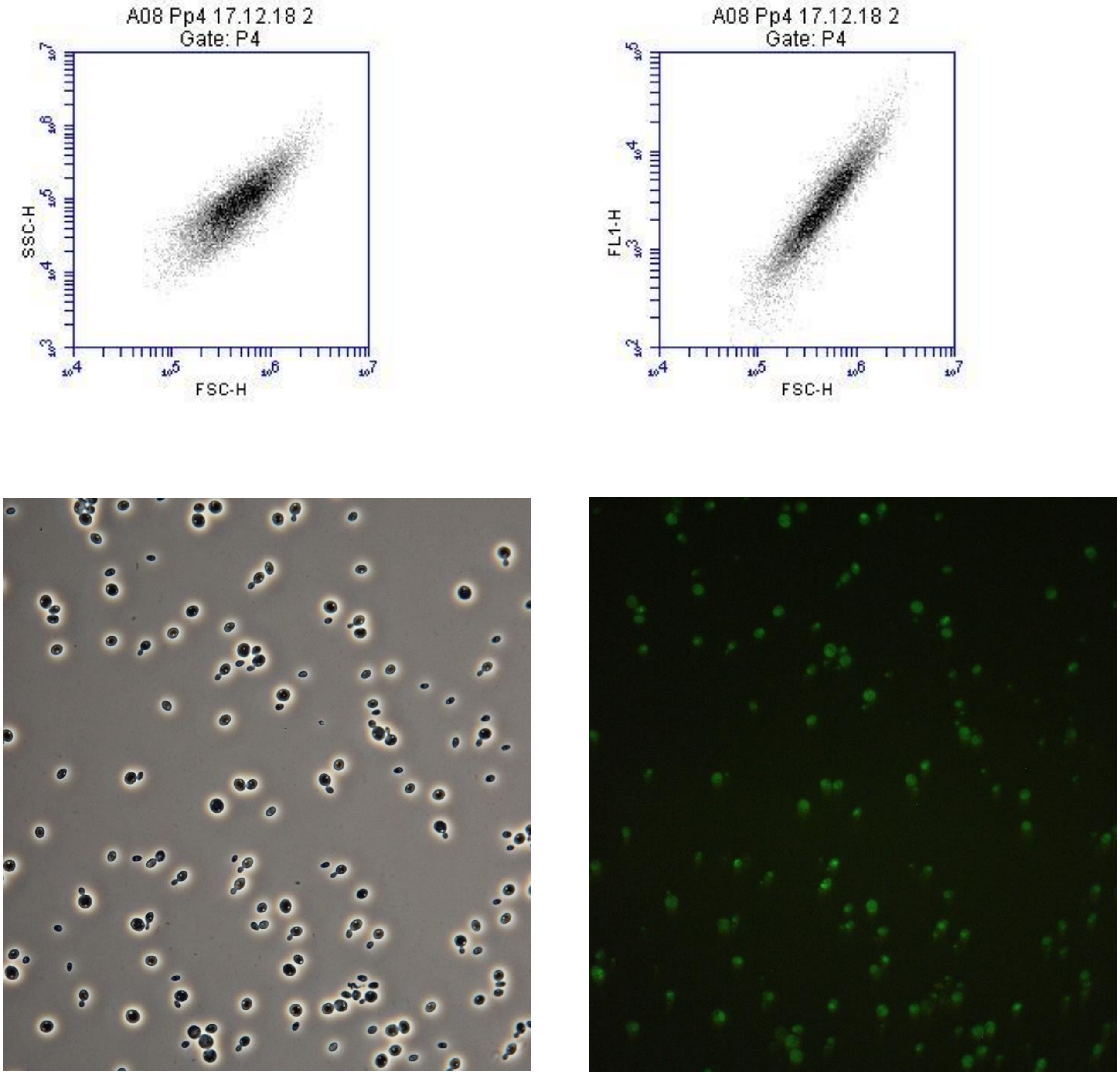

Supplementary Figure 7. Flow cytometry and microscopic analysis of the *P. pastoris* strain producing *Ec*PGA cultured in a shake flask.

5 mL of BMG was inoculated with a single colony of the strain producing *Ec*PGA (Pp4) and cultured overnight (30 °C, 200 rpm). The overnight-grown culture was used to inoculate 25 mL BMG to reach a starting OD_{600} 0.2 and incubated at 30 °C and 200 rpm. After 24 h, two methanol pulses (24 h, 48 h) were performed to reach and keep a methanol concentration of 15 g L^{-1}. After 60 h of the cultivation, a third pulse was performed to reach a methanol concentration of 30 g L^{-1}. After 120 h from the start of cultivation, the population was analyzed with flow cytometry (BD Accuri™ C6) and microscopy (light, fluorescence). (A) FSC-SSC dot plot, (B) FSC-FL1 dot plot, (C) light microscopy, (D) fluorescent microscopy.

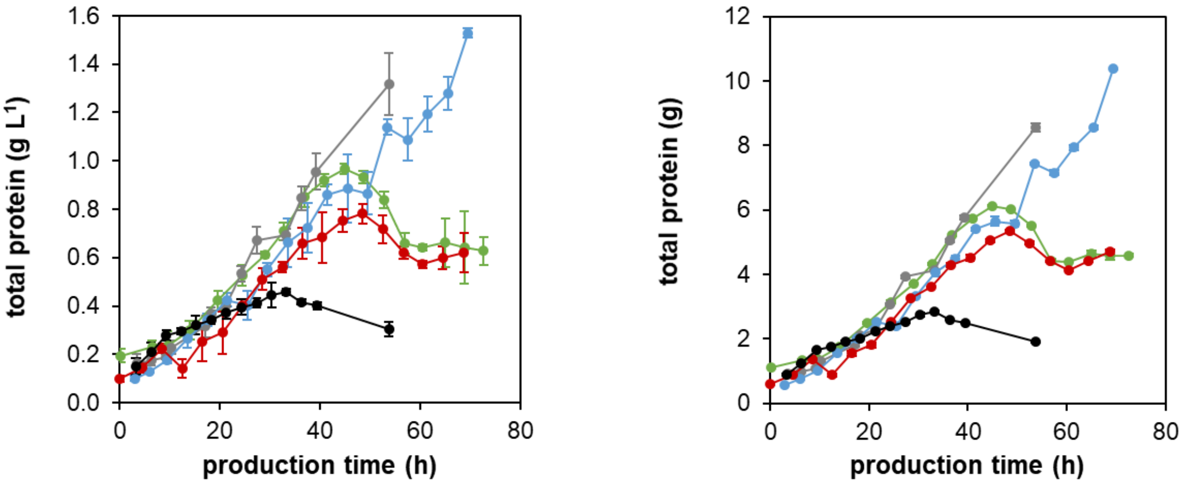

Supplementary Figure 8. Time course of the extracellular total protein concentration and mass during bioreactor fed-batch cultivations of *P. pastoris* strains.

The *P. pastoris* strains producing *Ec*PGA (green), *Ca*LB (blue) or *Tl*XynA (red), as well as the control strain producing only *Ec*PGA and not sfGFP (grey), and the control strain producing only sfGFP and no other recombinant protein (black), were cultured at 30 °C, pH 5.5 and a specific growth rate with methanol of 0.016 h^{-1}. The total protein concentration (g L^{-1} centrifuged medium) was assessed by Bradford assay in the centrifuged cultivation medium and recalculated to the volume of culture broth (g L^{-1}) (A) and to protein mass (g) (B).

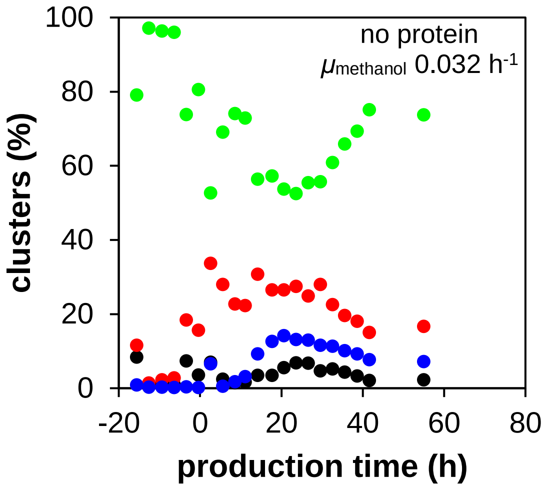

Supplementary Figure 9. Change of cell size and complexity, UPR up-regulation and viability during the cultivation of the *P. pastoris* non-producing control strain at μ 0.032 h^{-1}.

The *P. pastoris* non-producing control strain was cultured at 30 °C, pH 5.5 and at the specific growth rate of biomass with methanol at 0.032 h^{-1}. The four sub-populations identified with the PCA of the flow cytometry data were observed in all the cultivation processes: smaller and less complex viable cells with no UPR up-regulation (red ovals); bigger and more complex viable cells with no UPR up-regulation (green ovals); viable cells with up-regulated UPR (black ovals); and cells with an impaired viability (blue ovals).

1.2 Supplementary Tables

Supplementary Table 1. List of primers used in this work.

Name	Sequence
PP_8	GAATTCATCGATGATTTACCCATACGACGTACCAGATTACGCTTAACACGTCCGACGGCGGCCCACGGGTCCC<br>HA epitope tag, stop codon
PP_9	ACTAGTGGATCTGATAGCTTGCAAATTAAAGCCTTCGAGCGT
PP_10	CCGCCAGCTGAAGCTAGCAAAAGTTCAAAATCACCAACTGG
PP_11	ATCCACTAGTTCTAGCCCGGGCGGGATATCGTCCATTCCGACAGCATCGCCA
PP_30	GACGATATCCCGCCCAGAGCTCCTAGTGAAGACTTGAGAT
PP_31	CACTAGTTCTAGCCCTCTTGAGTGTTGGAATTGAAATTAA
PP_94	GACGATATCCCGCCCGGGCTAGAACTAGTGGATCCCCCCCGGAATGTCCAAGGGTGAAGAGCT
PP_95	ATGGGTAAATCATCGAGGATCCCTTATAAAGCTCGT
PP_98	TGTGCCGGGGGACGCCGAGGCCATCGAGGCACTGGATG
PP_99	GCGTCCCCCGGCACACTGGTGCGGTACCGGTAAGCCG
PP_108	ATCCACTAGTGGCCTGACATGGAGGCCCAGAATACCCTCC
PP_109	GCAGATCCGCGGCCGGATTACAACAGGTGTTGTCCTCTGAG
alphaMF-CALB_fw	GAGAAGAGAGAGGCCGAAGCTTTGCCTTCAGGTTCAGACCCA
CALB-AOX1TT_rev	CAAATGGCATTCTGACATCCTCTTGATTAAGGGGTAACGATTCCTGAGCAAG
PAOX1-XYL_fw	AGATCAAAAAACAACTAATTATTCGAAACGATGGTTGGTTTCACCCCTGTTG
XYL-AOX1TT_rev	CAAATGGCATTCTGACATCCTCTTGATTAACCGACGTCTGCCACGGTGATTC
q24	ATGTGTGGAGACGAAGCCAG
q25	GGCGTAATCCCAGAGGTGTT
q34	ATCGCCGGTTTGACTGTTCT
q35	CATCGAAGGTTCCTCCACCC

Supplementary Table 2. Maximum specific growth rate (μ_{max}) and maximum biomass/substrate yield ($Y_{x/s,\ max}$) with glycerol and methanol of the strains used in this work.

The parameters μ_{max} and $Y_{x/s,\ max}$ were assessed during batch cultivations in bioreactor with 30 g L^{-1} glycerol and 15 g L^{-1} methanol.

	**Pp1 (control)**	**Pp4**	**Pp5 (control)**	**Pp10**	**Pp14**
$\mu_{max,\ glycerol}$ **(h^{-1})**	0.224 ± 0.035	0.229 ± 0.024	0.225 ± 0.014	0.263 ± 0.011	0.226 ± 0.009
$Y_{x/s,\ max,\ glycerol}$ **($g\ g^{-1}$)**	0.704 ± 0.030	0.622 ± 0.034	0.628 ± 0.065	0.702 ± 0.027	0.683 ± 0.021
$\mu_{max,\ methanol}$ **(h^{-1})**	0.042 ± 0.026	0.056 ± 0.034	0.052 ± 0.025	0.065 ± 0.016	0.060 ± 0.010
$Y_{x/s,\ max,\ methanol}$ **($g\ g^{-1}$)**	0.245 ± 0.031	0.313 ± 0.131	0.279 ± 0.097	0.344 ± 0.075	0.306 ± 0.046
